# Supplementary material for: N-Alkyl Sulfamates as a New Class of nsP2 Cysteine Protease Inhibitors with Broad Spectrum Antialphaviral Activity
Source: bioRxiv. 2025 Jul 4:2025.06.30.662352. Preprint. [Version 1] doi: 10.1101/2025.06.30.662352 (PMC12236718; doi:10.1101/2025.06.30.662352)
Supplement: Supplement 1 [file media-1.pdf]

SUPPORTING INFORMATION

N-Alkyl Sulfamates as a New Class of nsP2 Cysteine Protease Inhibitors with Broad Spectrum Antialphaviral Activity

Anirban Ghoshal[†,#], John D. Sears[§,#], Mohammad Anwar Hossain[†,#], Edwin G. Tse[†,#], Stefanie Howell[†,#], Jane E. Burdick[#,⊥], Noah L. Morales[#,⊥], Sabian A. Martinez[#,⊥], Isabella Law[#,⊥], Zachary J. Streblow[#,ǁ], Daniel N. Streblow[#,ǁ], Rafael Miguez Couñago[†,#], Nathaniel J. Moorman[§,#], Mark T. Heise[⊥,§,#], and Timothy M. Willson[†,#,]*

[†]Structural Genomics Consortium, UNC Eshelman School of Pharmacy, University of North Carolina at Chapel Hill, Chapel Hill, NC 27599, USA.

[#]READDI AViDD Center, University of North Carolina at Chapel Hill, Chapel Hill, NC 27599, USA.

[§]Department of Microbiology and Immunology, University of North Carolina at Chapel Hill, Chapel Hill, NC, 27599, USA

[⊥]Department of Genetics, University of North Carolina at Chapel Hill, Chapel Hill, NC, 27599, USA

[ǁ]Vaccine & Gene Therapy Institute, Oregon Health & Science University, Beaverton, OR, 97006, USA

*Correspondence: tim.willson@unc.edu

Table of Contents

		Page
Table S1	Aqueous solubility	S2
Table S2	Inhibitory effects of **5** on human and viral cysteine proteases	S3
Figure S1	Chemical stability of **5** at room temperature	S4
Figure S2	DLS data for **5**	S5
Figure S3	GSH stability of **5**	S6
Figure S4	Cell toxicity data upon 48 h exposure of **5**	S7
Figure S5	MS fragmentation of metabolites **M1–13** from mouse hepatocytes	S8–S12
Figures S6–S45	NMR spectra of final compounds	S13–S32
Figures S46–S66	HPLC analyses	S33–S42

Table S1. **Aqueous solubility**[a]

Compound	Aqueous Solubility (µM)[a]
**1**	150
**2**	50
**3**	5
**4**	90
**5**	70
**6a**	20
**6b**	30
**6c**	140
**6d**	40
**6e**	40
**6f**	190
**7a**	1
**7b**	190
**7c**	1
**7d**	150
**8a**	220
**8b**	220
**8c**	220
**9a**	150
**9b**	150
**9c**	70

[a]Kinetic solubility of a 10 mM DMSO solution at pH 7.4.

Table S2. Inhibitory effects of **5** on human and viral cysteine proteases

Enzyme	Substrate	Control	% Inhibition			
			**5** (10 μM)	**Reference** (0.1 x IC_{50})[a]	**Reference** (1 x IC_{50})[a]	**Reference** (10 x IC_{50})[a]
Cathepsin L	5 μM Z-Leu-Arg-AMC	E-64	14	7	51	96
USP7	100 nM Ub-AMC	Ub-Aldehyde	3	2	37	95
UCHL1	100 nM Ub-AMC	Ub-Aldehyde	8	2	33	92
3CLPro	3CL Pro Substrate	GC376	8	11	47	95

[a]IC_{50} of the control nsP2 protease inhibitor.

Figure S1. Chemical stability of **5** at room temperature

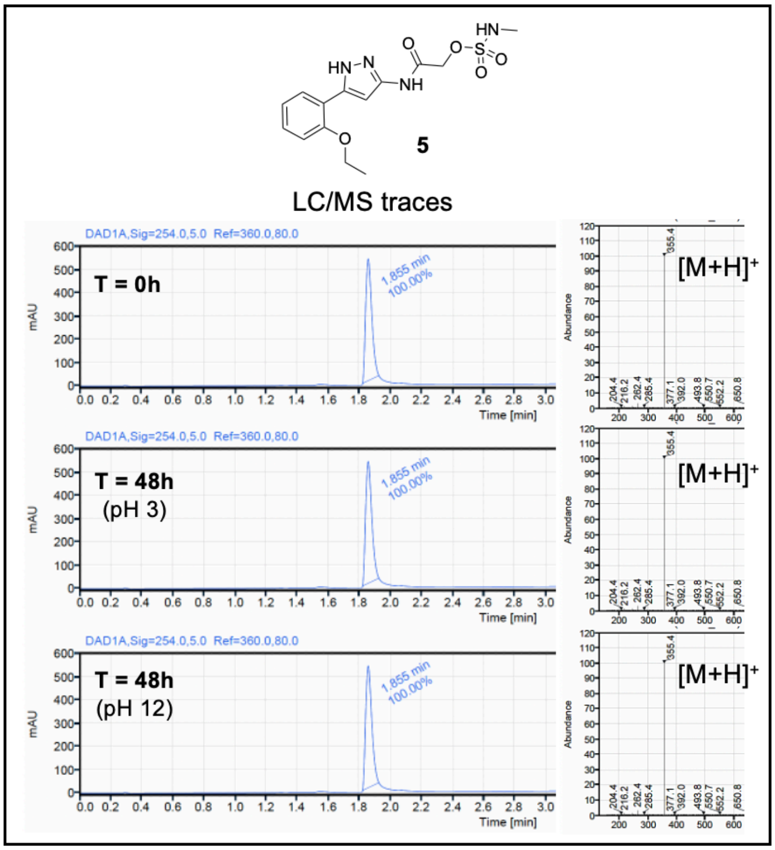

Figure S2. DLS data for **5**

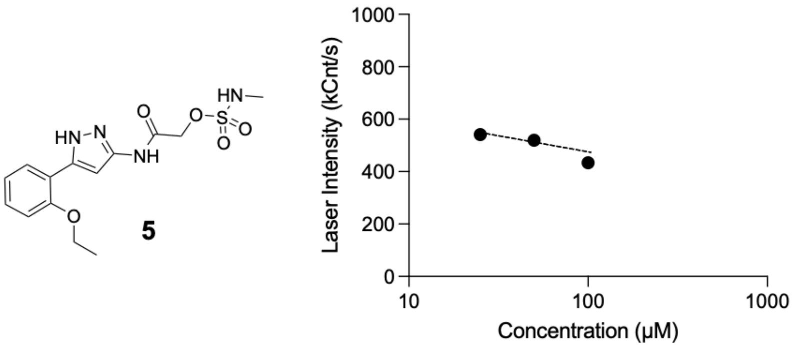

.

Dynamic Light Scattering (DLS) Assay. Aggregation behavior of sulfamate **5** was determined by DLS in duplicate at 25 ºC in 25 mM HEPES buffer (pH 7.4) containing 0.003% Tween-20 and 1 mM DTT, using a 10 mM stock solution containing 2% DMSO. Light scattering was measured using a DynaPro Plate Reader III. Buffer with 2% DMSO control produced an average laser intensity of 858 kCnts/s.

Figure S3. GSH stability of **5**

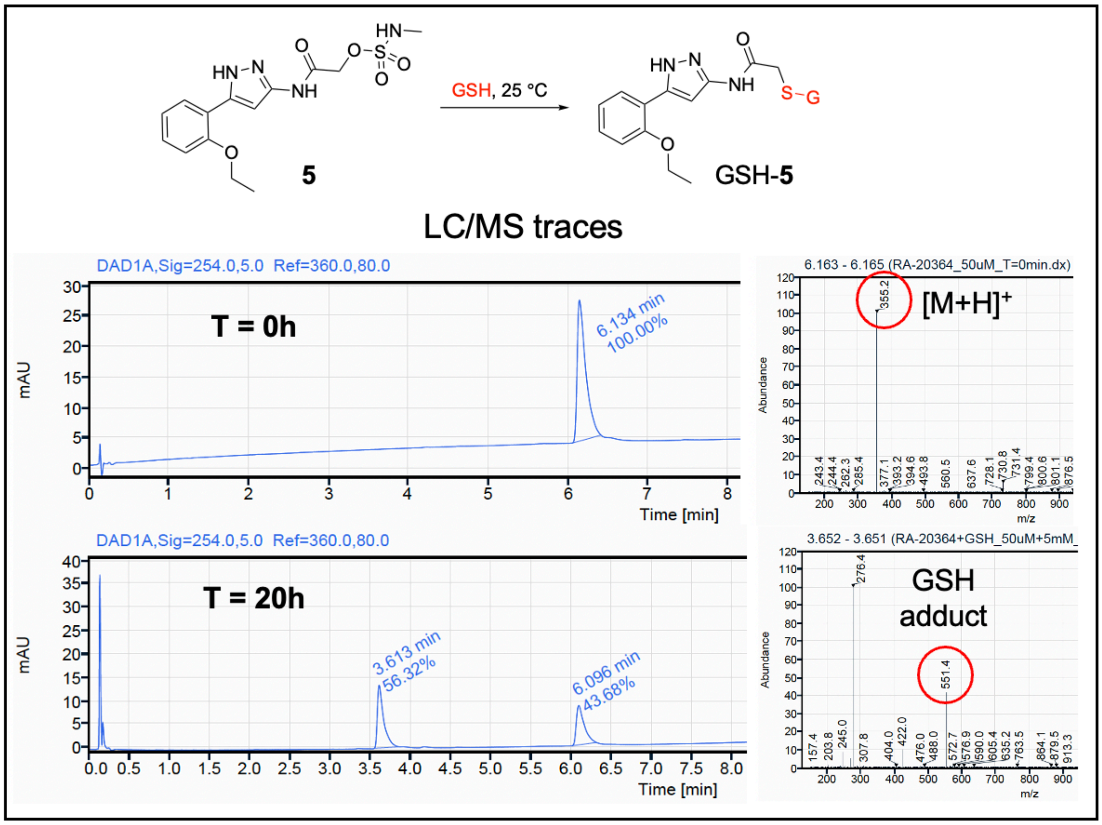

Method for GSH stability. A 10 mM solution of GSH (Sigma Aldrich Cat# G4251 (St. Louis, MO, USA)) was prepared in a pH 7.4 phosphate buffer. A 10 mM DMSO solution of Compound **5** was diluted in phosphate buffer to give a solution at 100 µM with 1% DMSO. At time zero (t = 0), 50 µL of the 100 µM test compound solution was added to an Eppendorf tube containing 50 µL phosphate buffer and 50 µL of 10 mM GSH solution. The final concentrations of the compound and GSH were maintained at 50 µM and 5 mM, respectively. The Eppendorf tube was vortexed, and then the sample was transferred to a high-recovery autosampler vial for LCMS analysis. Analysis was performed every 1 h interval, and the percentage of GSH adduct formation was calculated using Agilent LCMS software (OpenLab CDS Version 2.7).

Figure S4. Cell toxicity data upon 48 h exposure of **5**

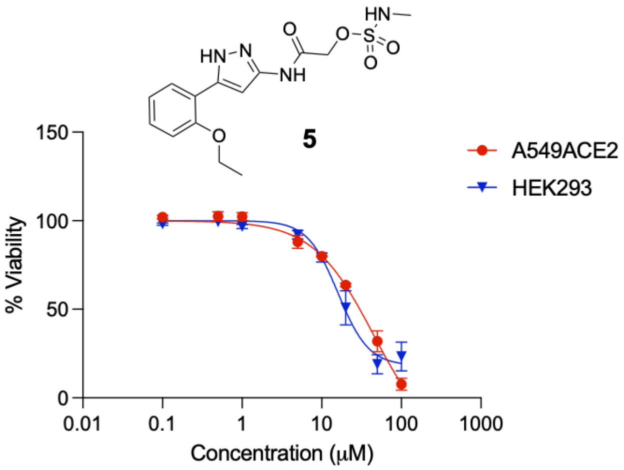

Cell viability assay. Cells were plated on a 384 well plate at a seeding density of 2000 cells/well for A549ACE2 (in DMEM high-glucose, 10% fetal bovine serum, 1X non-essential amino acids, and 2 mM L-glutamine) and HEK293 (in DMEM high-glucose, 10% fetal bovine serum) and allowed to adhere overnight. Cells were treated with dose response of compound, 1% DMSO (negative control), or 10% DMSO (positive control) for 48 h. CellTiter-Glo reagent (Promega) was added to each well and luminescence was detected on GloMax plate reader. There were two biological replicates containing treatments in quadruplicate. Cell viability was calculated using the following formula: ((raw RLU value – average of positive control wells) / (average of negative control wells – average of positive control wells))*100. Plots were generated using GraphPad Prism.

Figure S5. MS fragmentation of metabolites **M1–13** of **5** from mouse hepatocytes

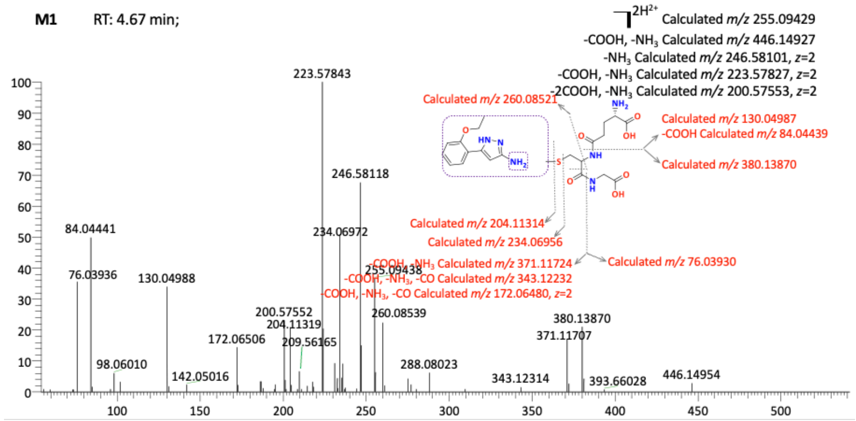

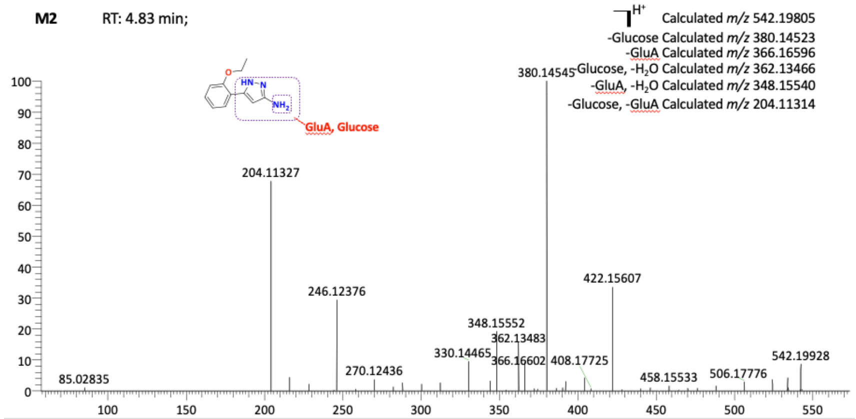

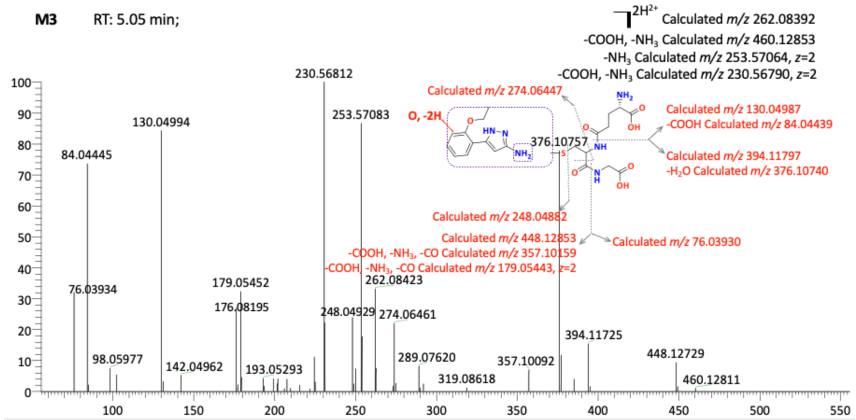

M4 RT: 5.07 min;

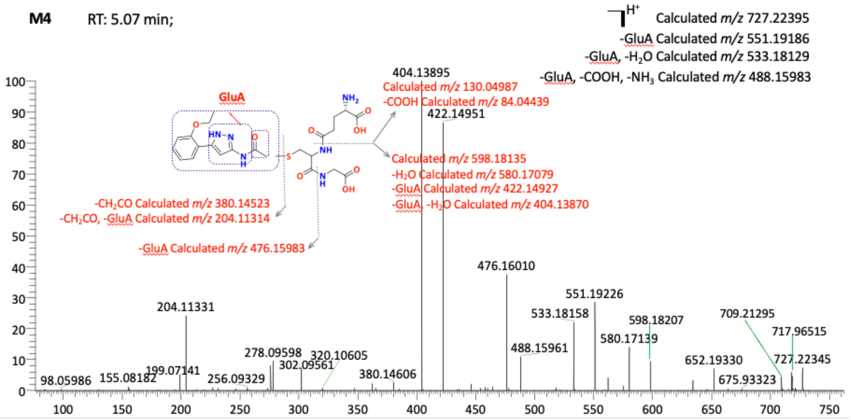

M5 RT: 5.09 min;

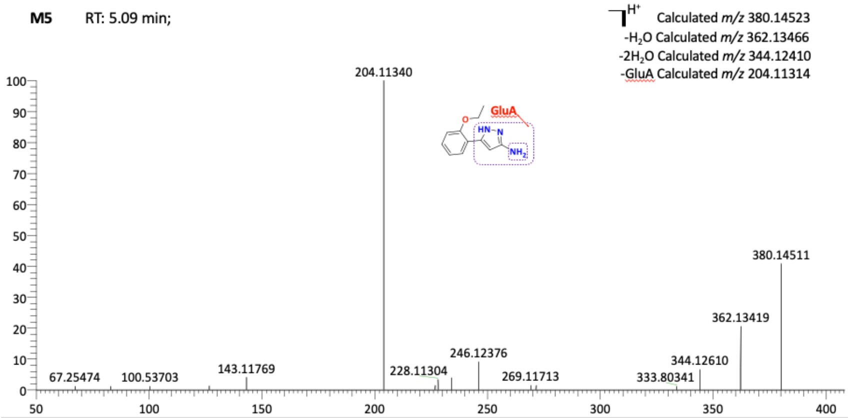

M6 RT: 5.36 min;

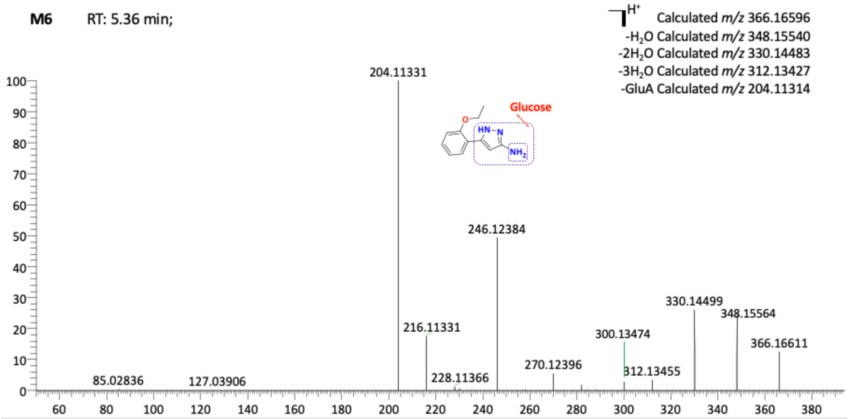

M7 RT: 5.71 min;

H⁺ Calculated *m/z* 503.10785
-GluA Calculated *m/z* 327.07577

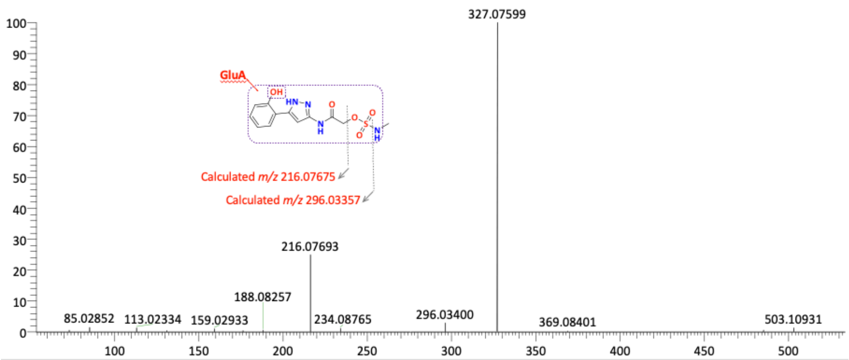

M8 RT: 5.87 min;

H⁺ Calculated *m/z* 204.11314

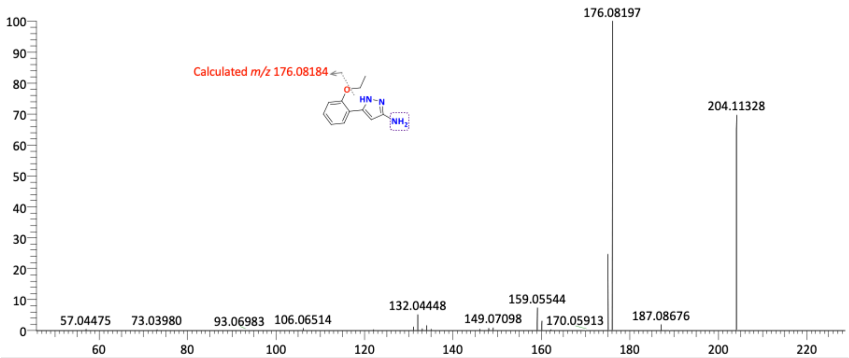

M9 RT: 5.99 min;

2H²⁺ Calculated *m/z* 276.09957
-NH_3 Calculated *m/z* 267.58629, *z*=2
-COOH, -NH_3 Calculated *m/z* 244.58355, *z*=2

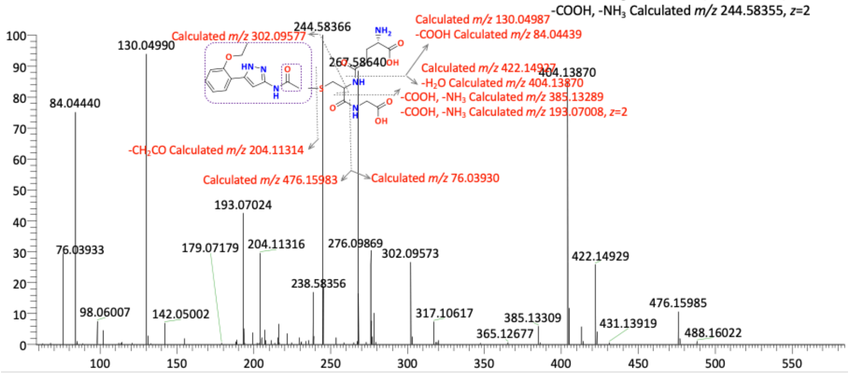

M10 RT: 6.38 min;

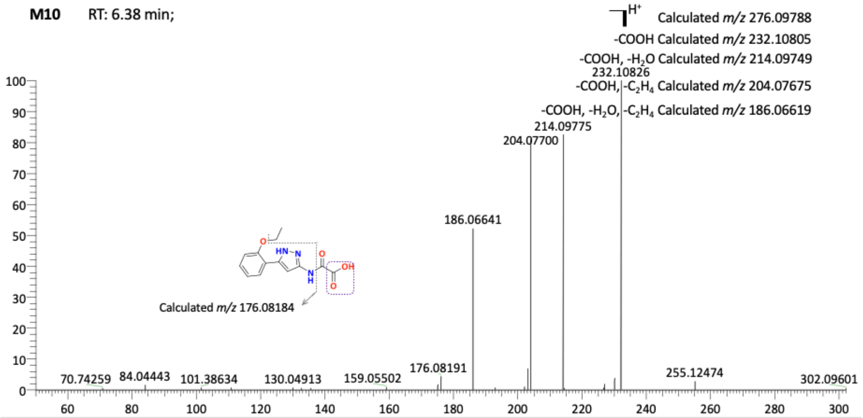

M11 RT: 6.39 min;

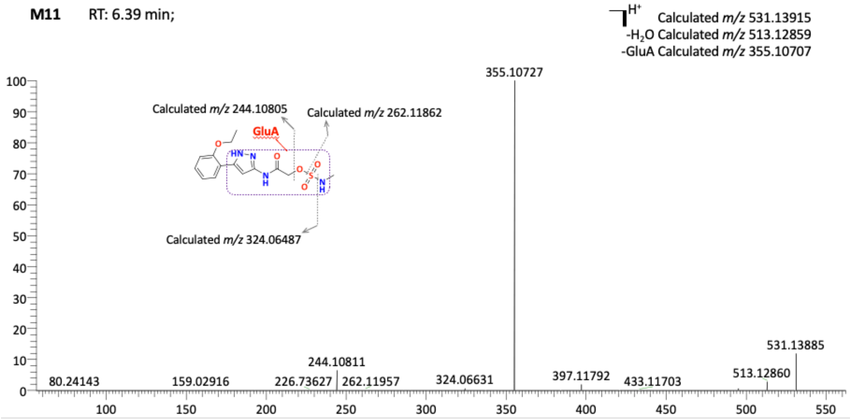

M12 RT: 7.07 min;

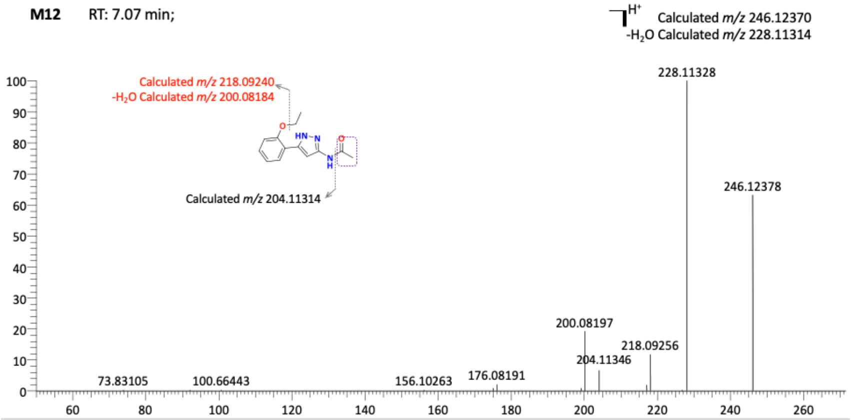

M13 RT: 7.42 min;

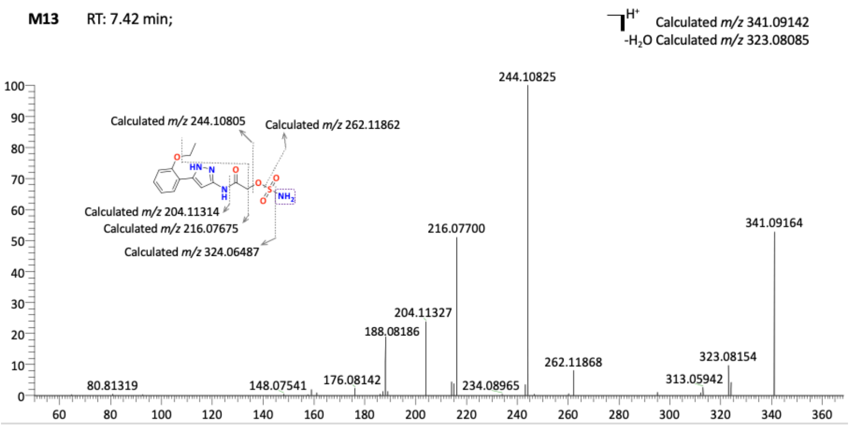

NMR spectra of final compounds

Figure S6: ^{1}H NMR (400 MHz, DMSO-d_6) for **2**

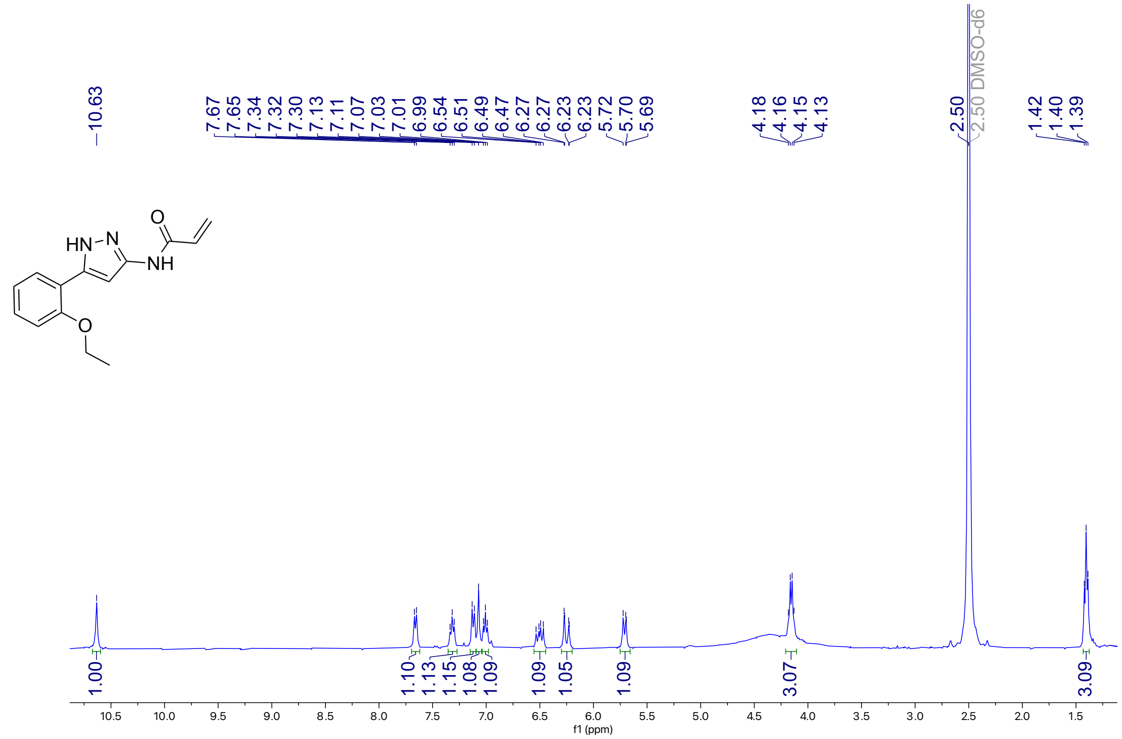

Figure S7: ^{13}C NMR (101 MHz, DMSO-d_6) for **2**

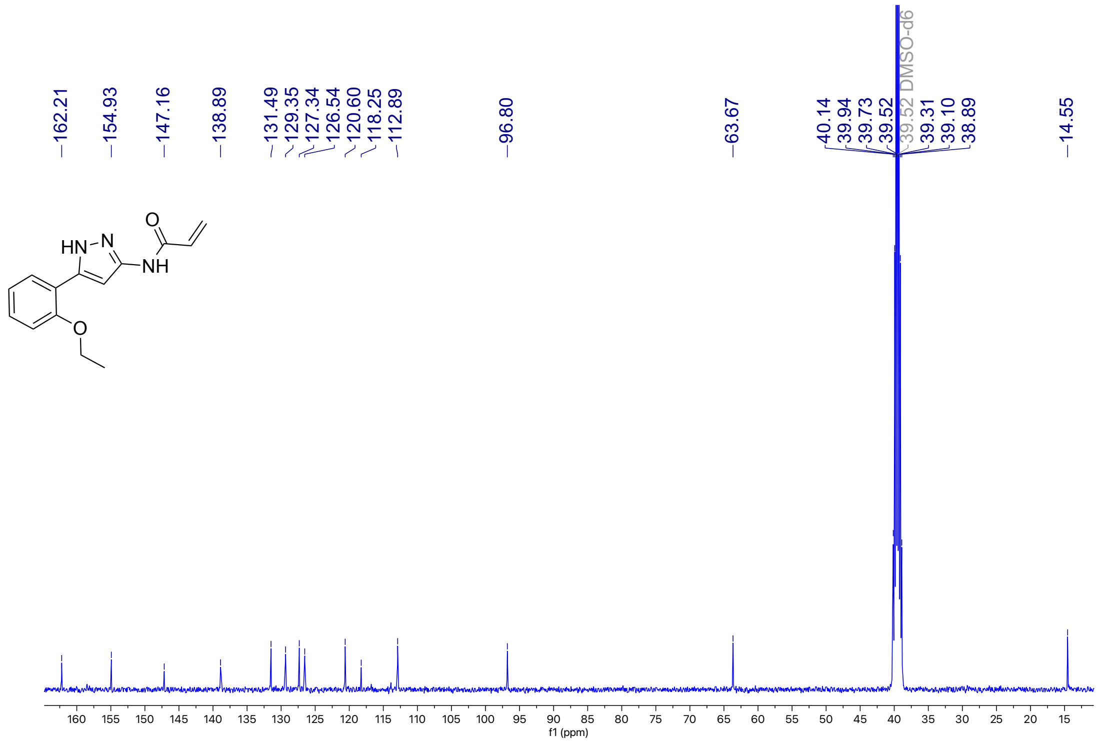

Figure S8: ^{1}H NMR (400 MHz, DMSO-d_6) for **3**

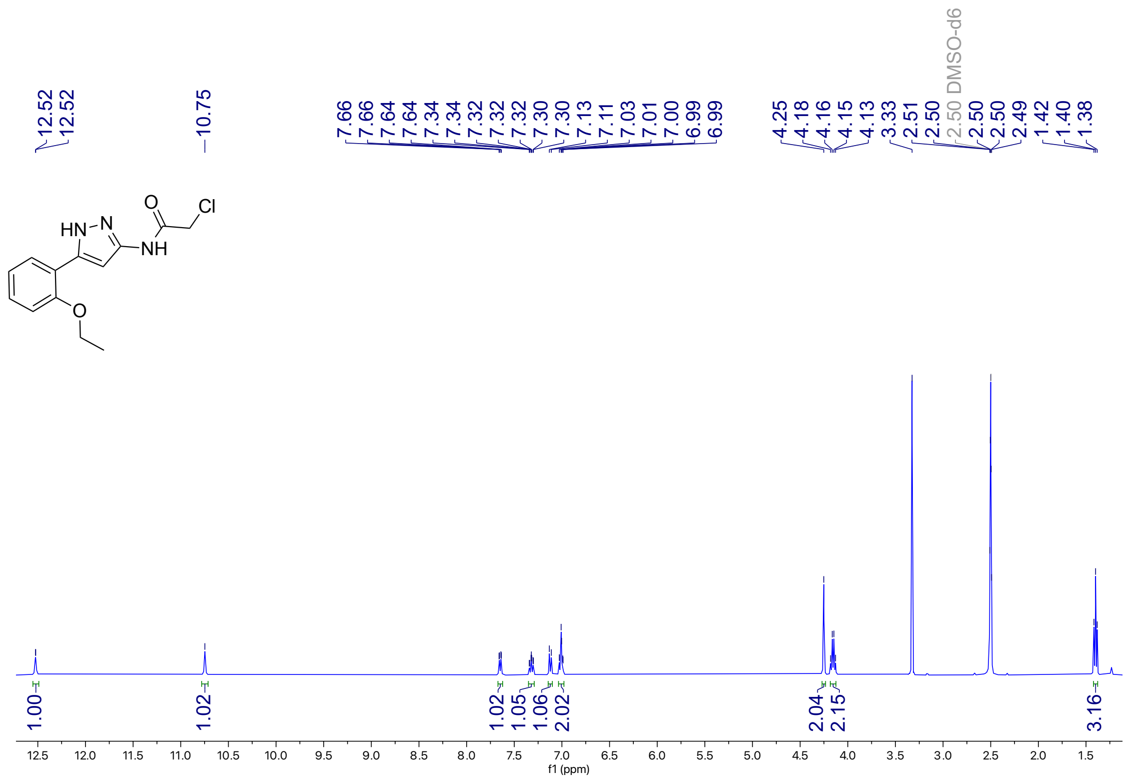

Figure S9: ^{13}C NMR (101 MHz, DMSO-d_6) for **3**

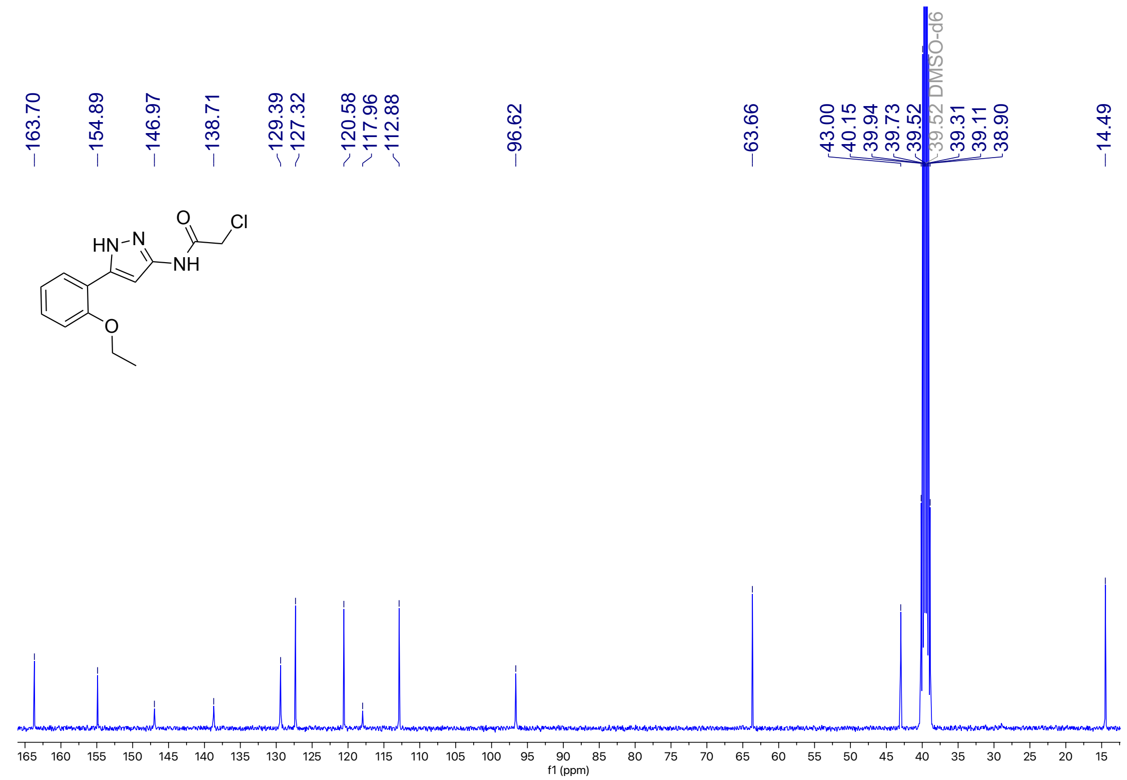

Figure S10: ^{1}H NMR (500 MHz, DMSO-d_6) for **4**

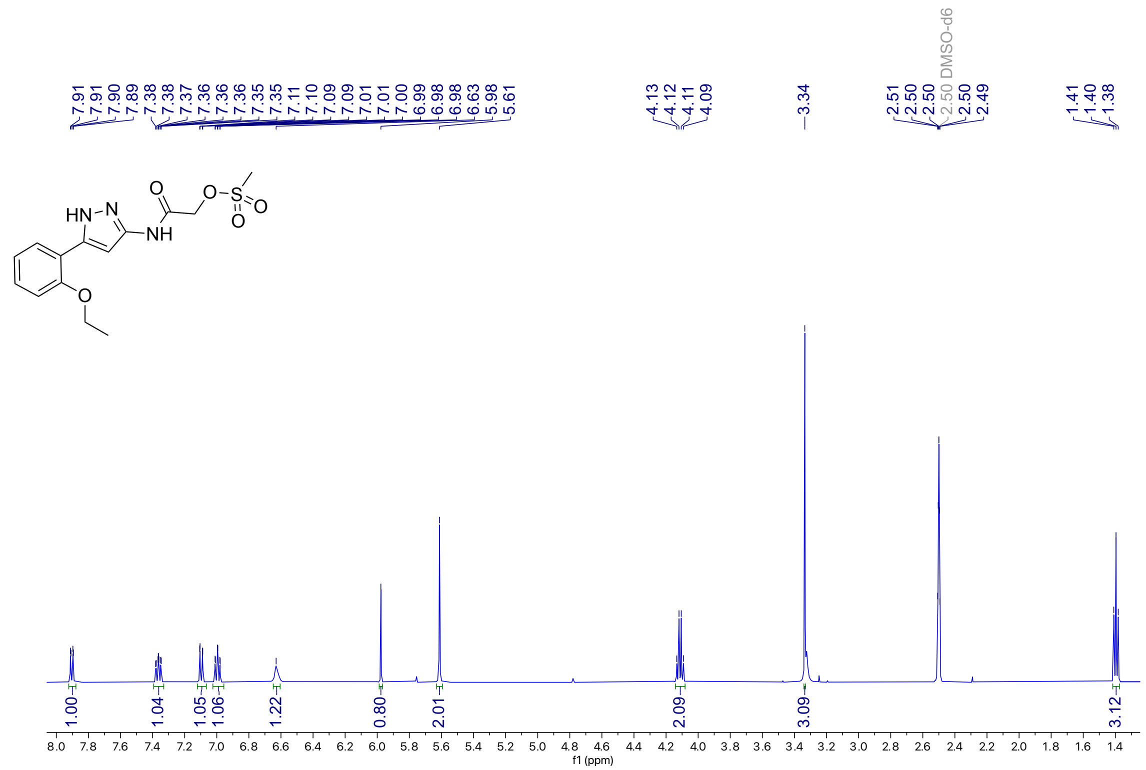

Figure S11: ^{13}C NMR (126 MHz, DMSO-d_6) for **4**

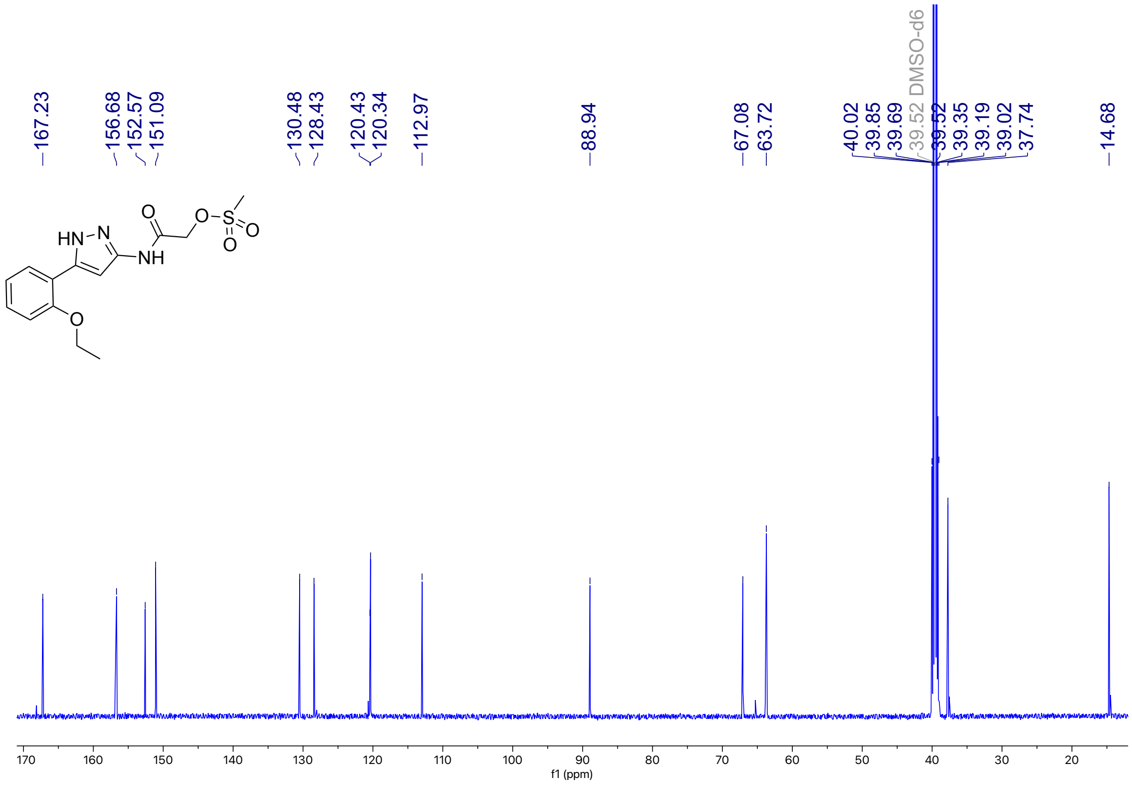

Figure S12: ^{1}H NMR (401 MHz, DMSO-d_6) for **5**

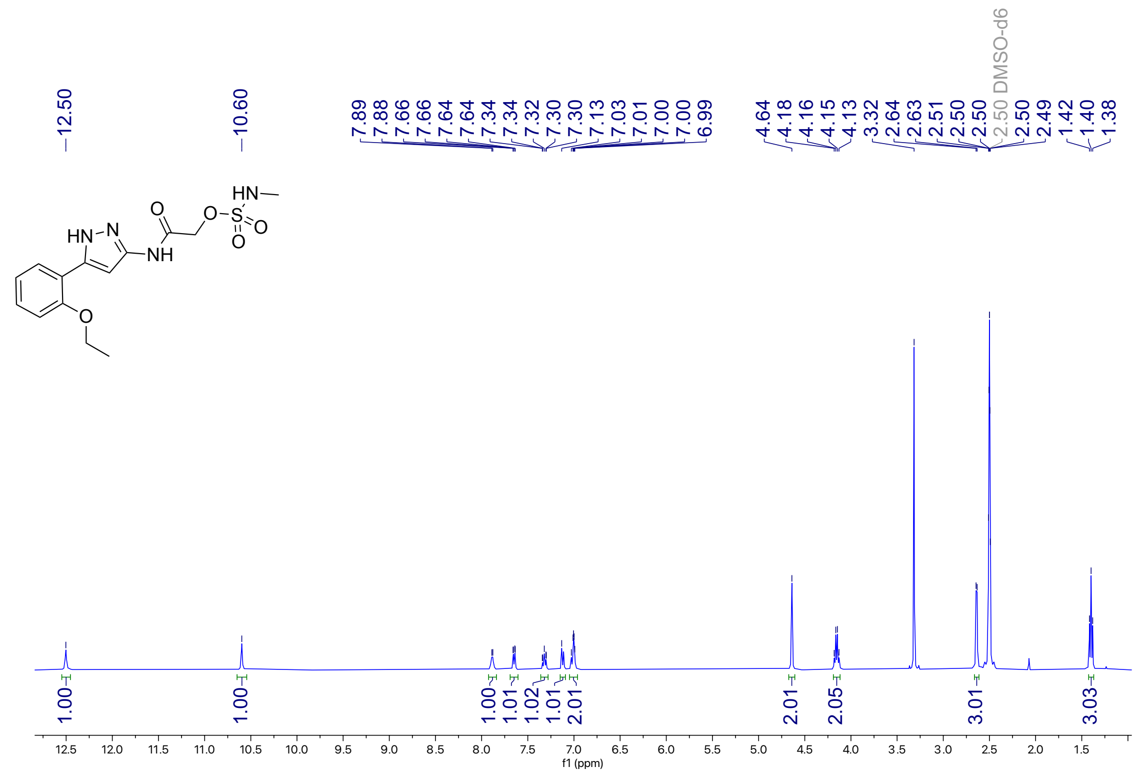

Figure S13: ^{13}C NMR (101 MHz, DMSO-d_6) for **5**

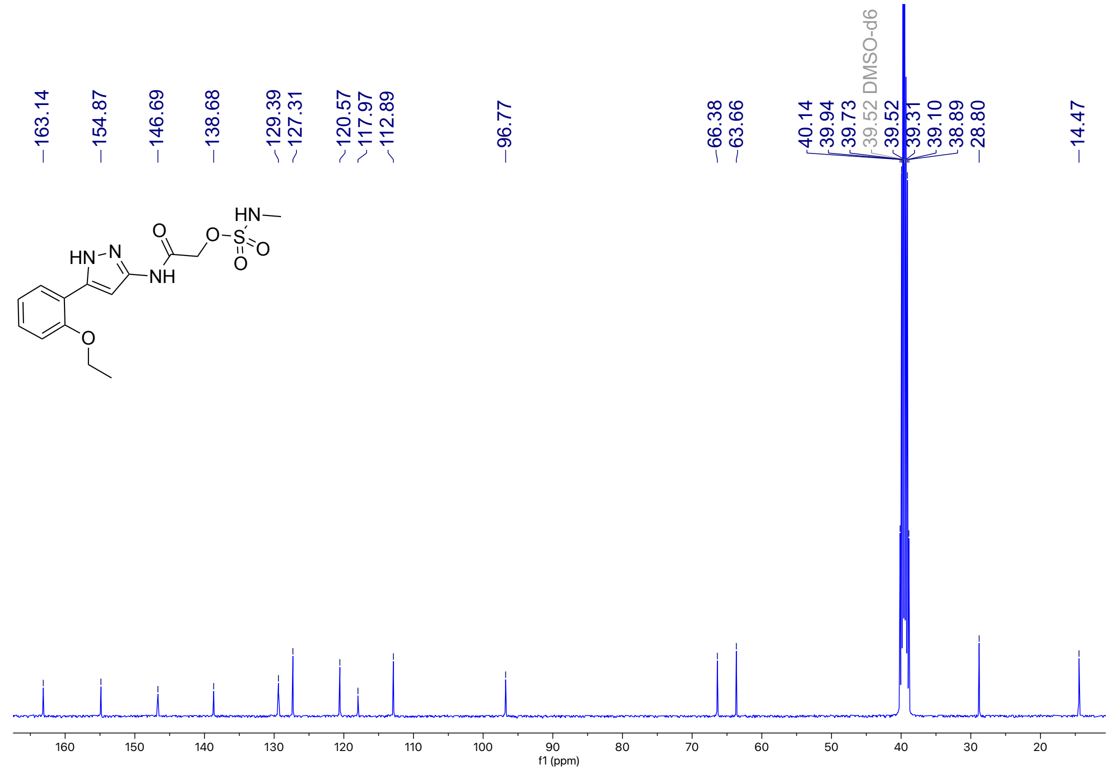

Figure S14: ^{1}H NMR (400 MHz, DMSO-d_6) for **6a**

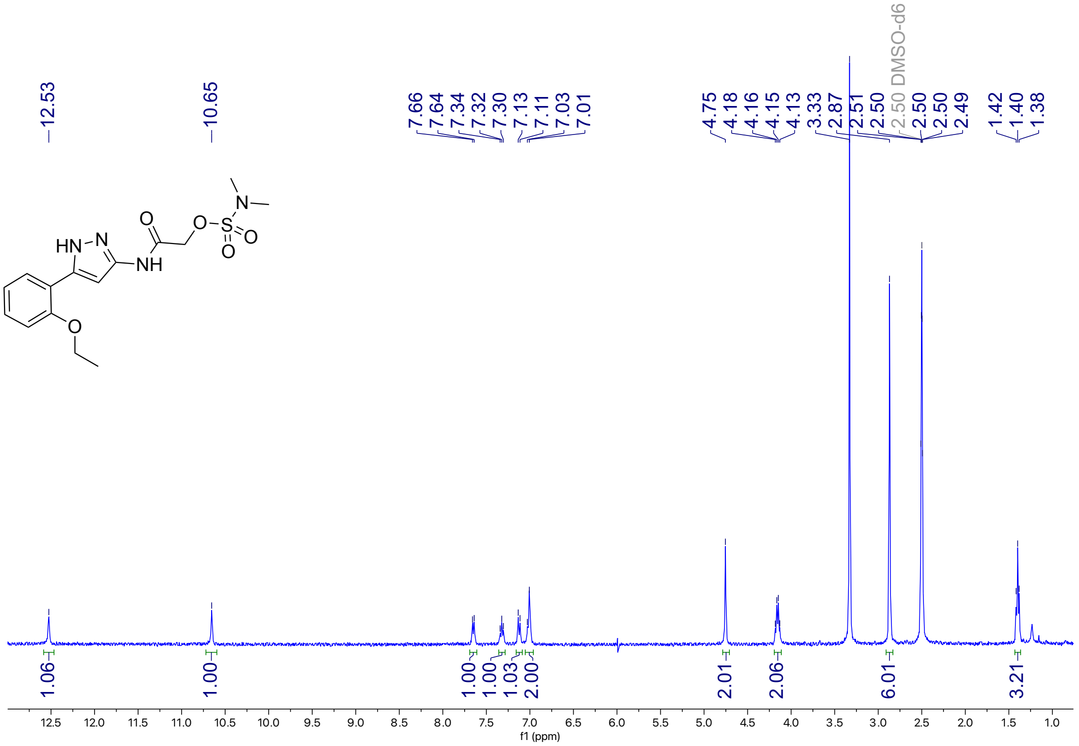

Figure S15: ^{13}C NMR (101 MHz, DMSO-d_6) for **6a**

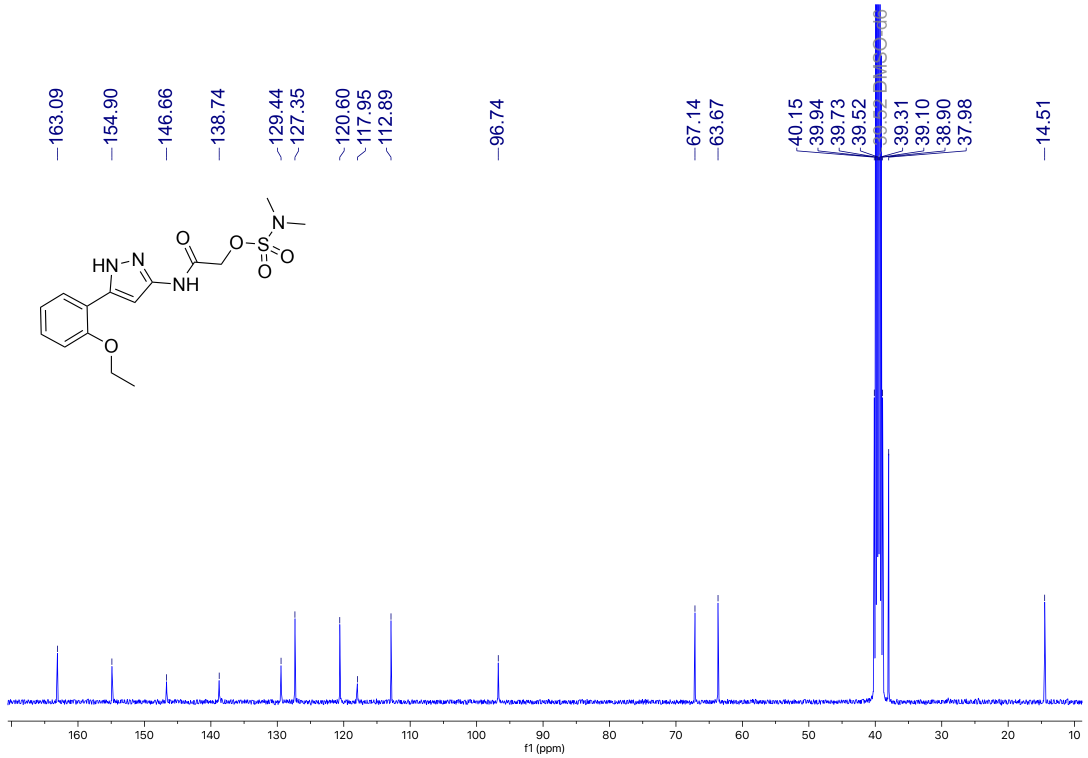

Figure S16: ^{1}H NMR (400 MHz, DMSO-d_6) for **6b**

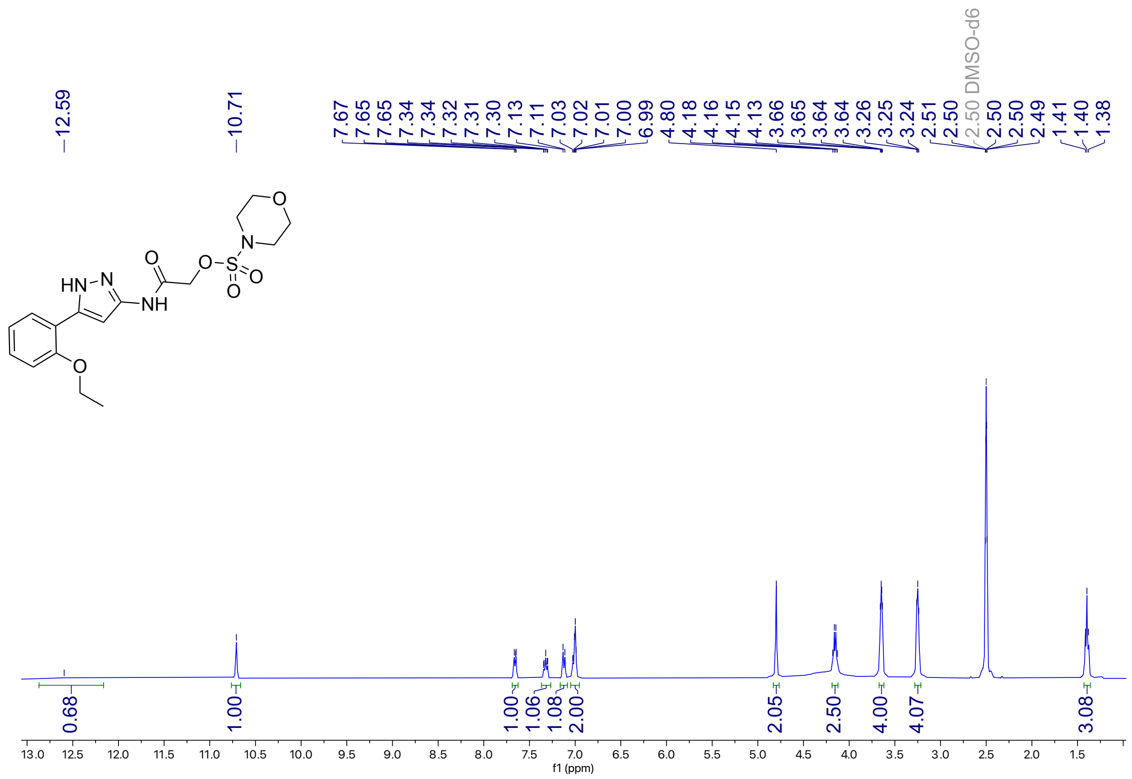

Figure S17: ^{13}C NMR (214 MHz, DMSO-d_6) for **6b**

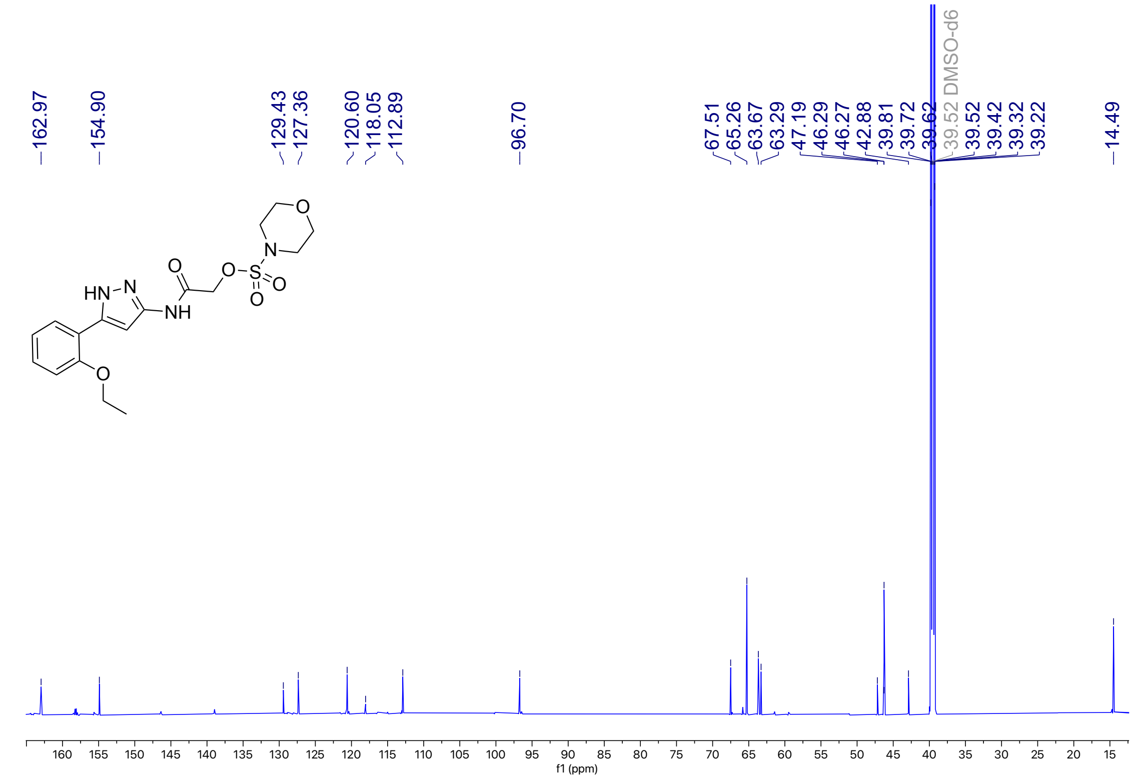

Figure S18: ^{1}H NMR (401 MHz, DMSO-d_6) for **6c**

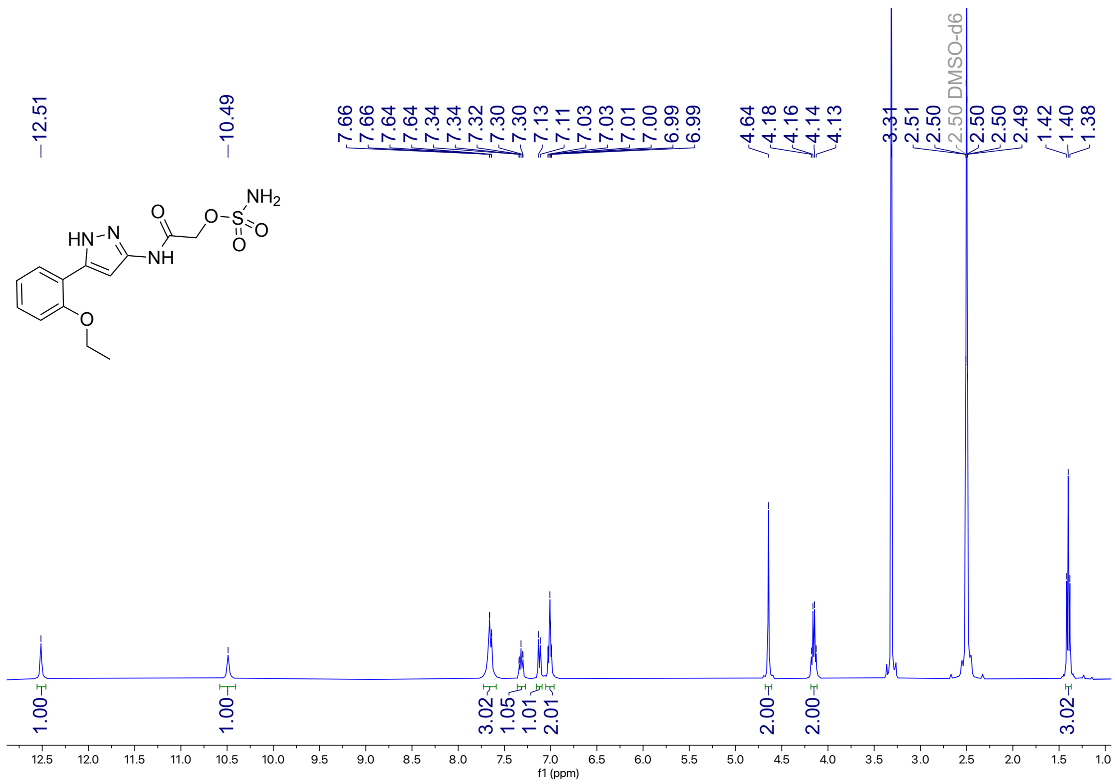

Figure S19: ^{13}C NMR (101 MHz, DMSO-d_6) for **6c**

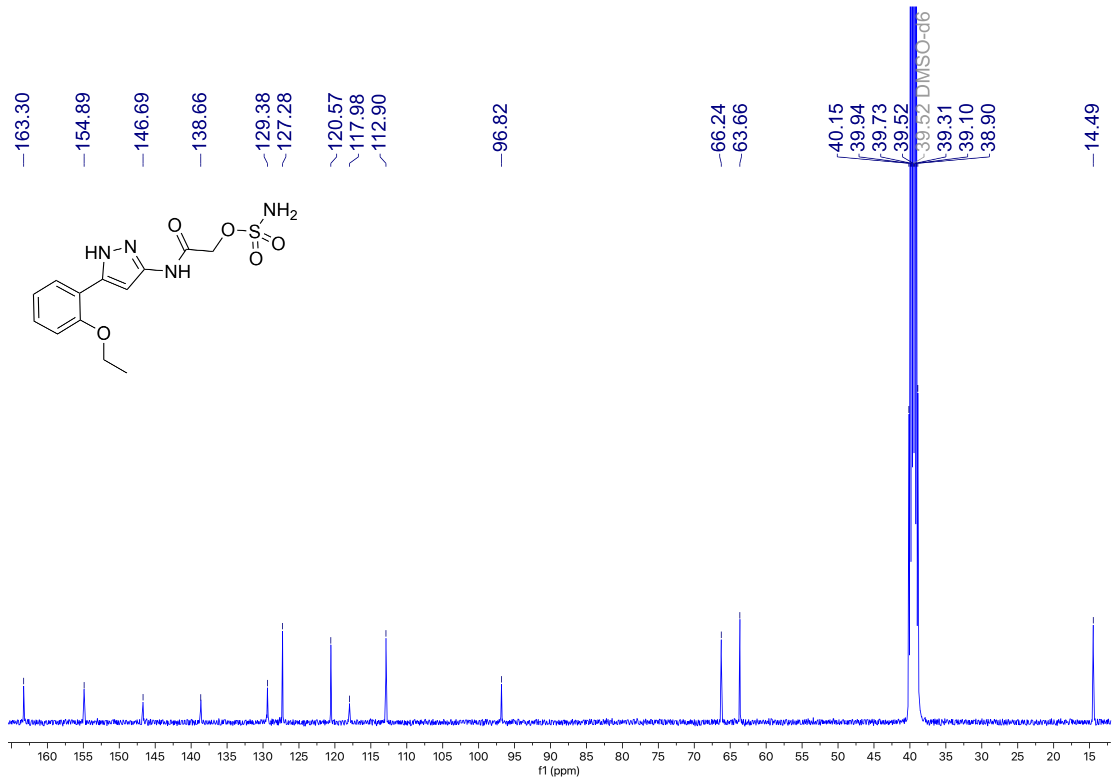

Figure S20: ^{1}H NMR (400 MHz, DMSO-d_6) for **6d**

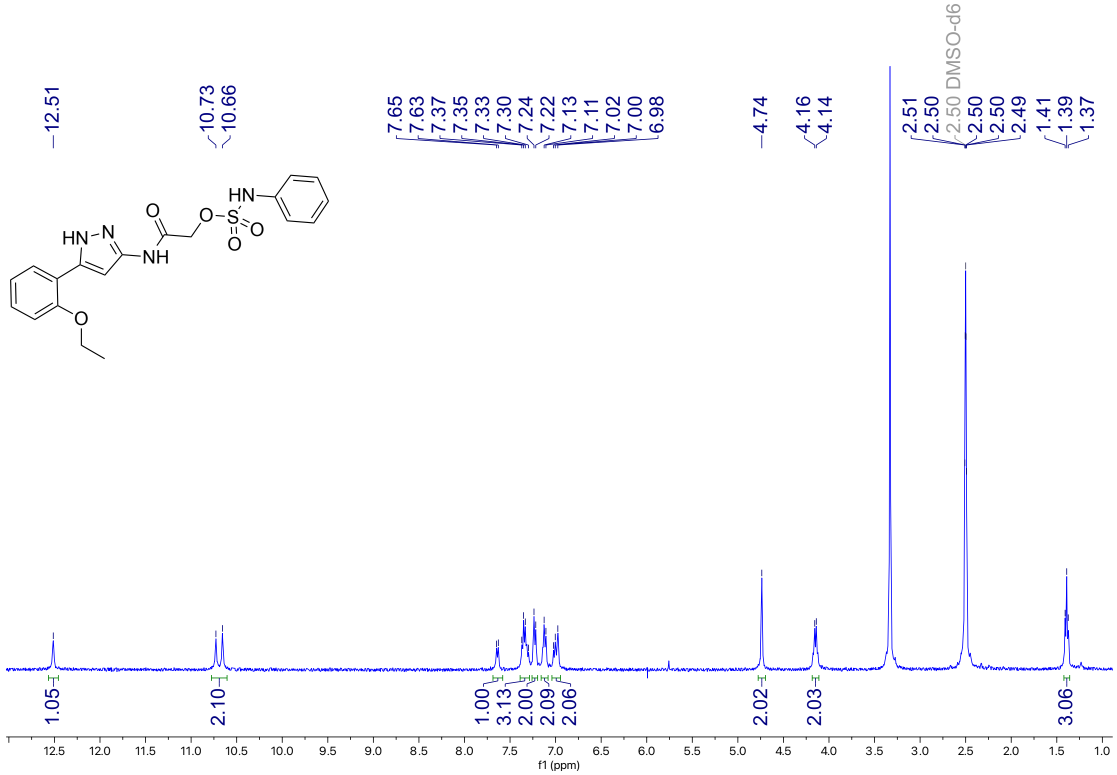

Figure S21: ^{13}C NMR (101 MHz, DMSO-d_6) for **6d**

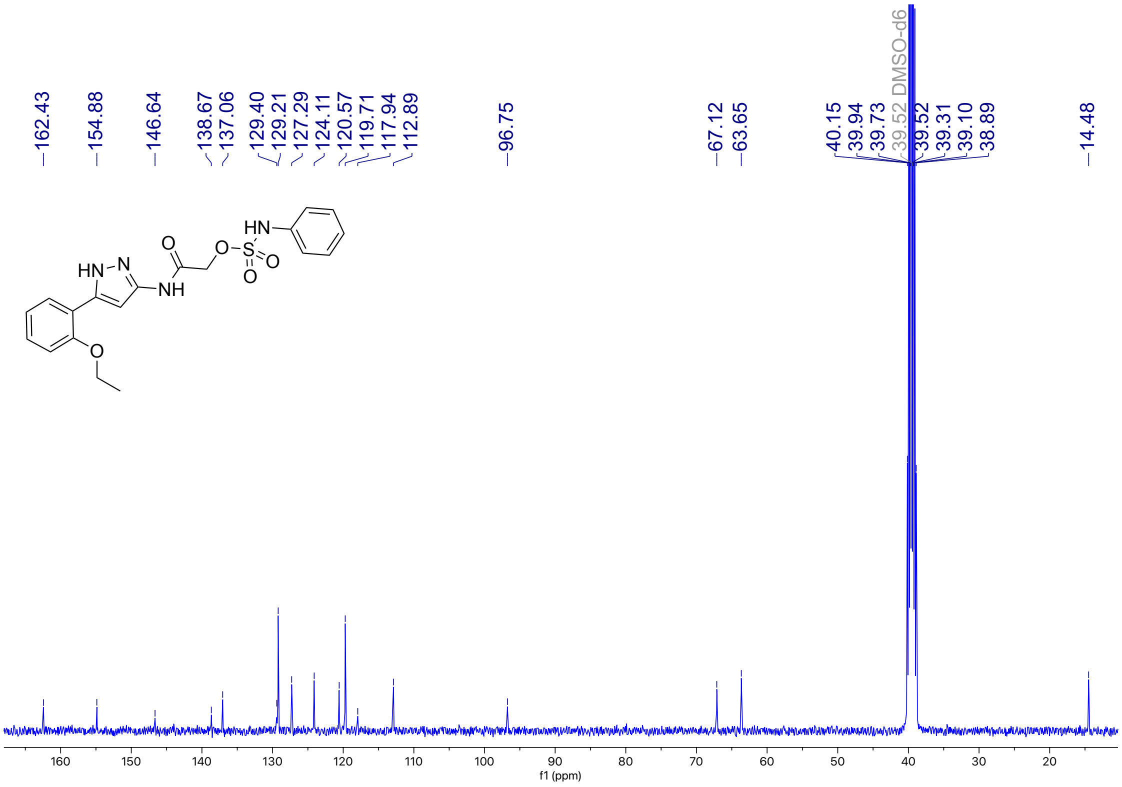

Figure S22: ^{1}H NMR (400 MHz, DMSO-d_6) for **6e**

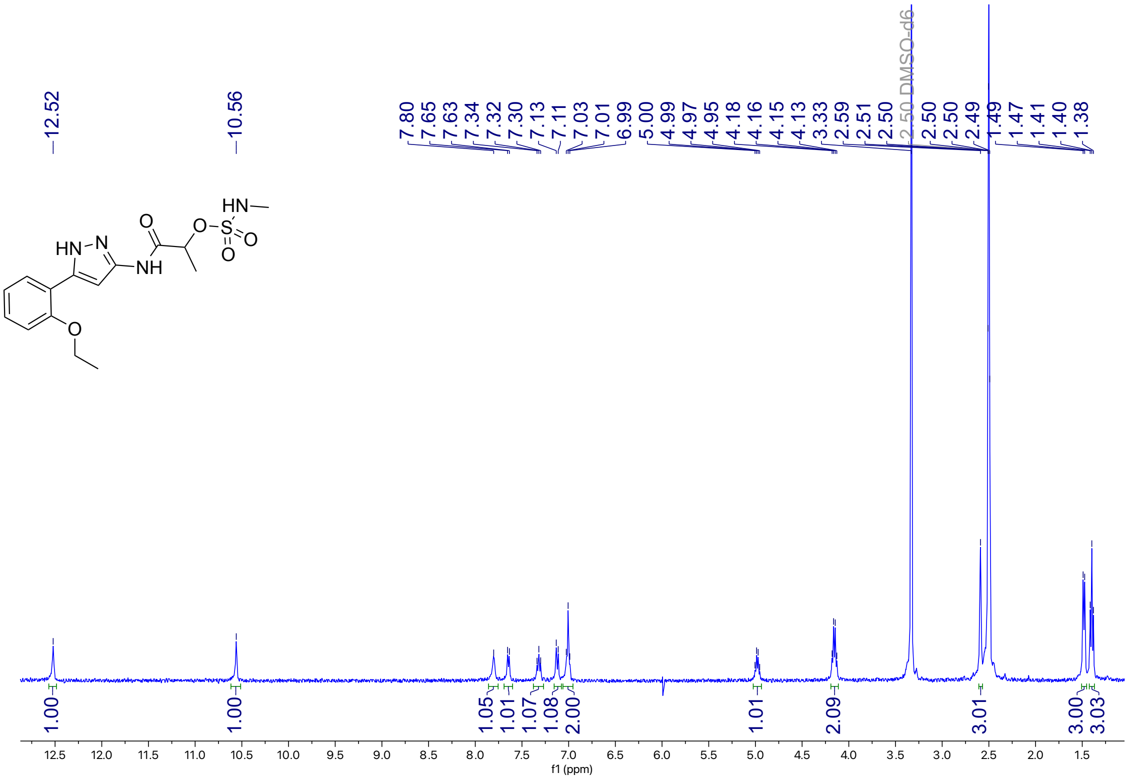

Figure S23: ^{13}C NMR (101 MHz, DMSO-d_6) for **6e**

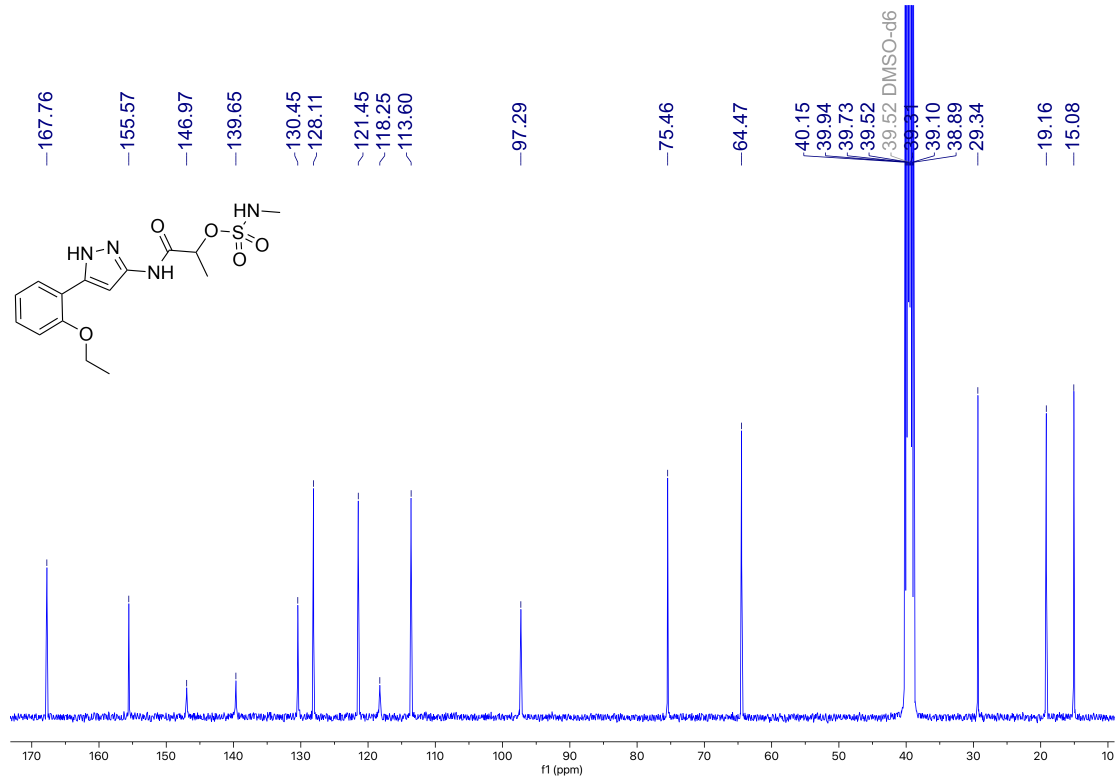

Figure S24: ^{1}H NMR (401 MHz, DMSO-d_6) for **6f**

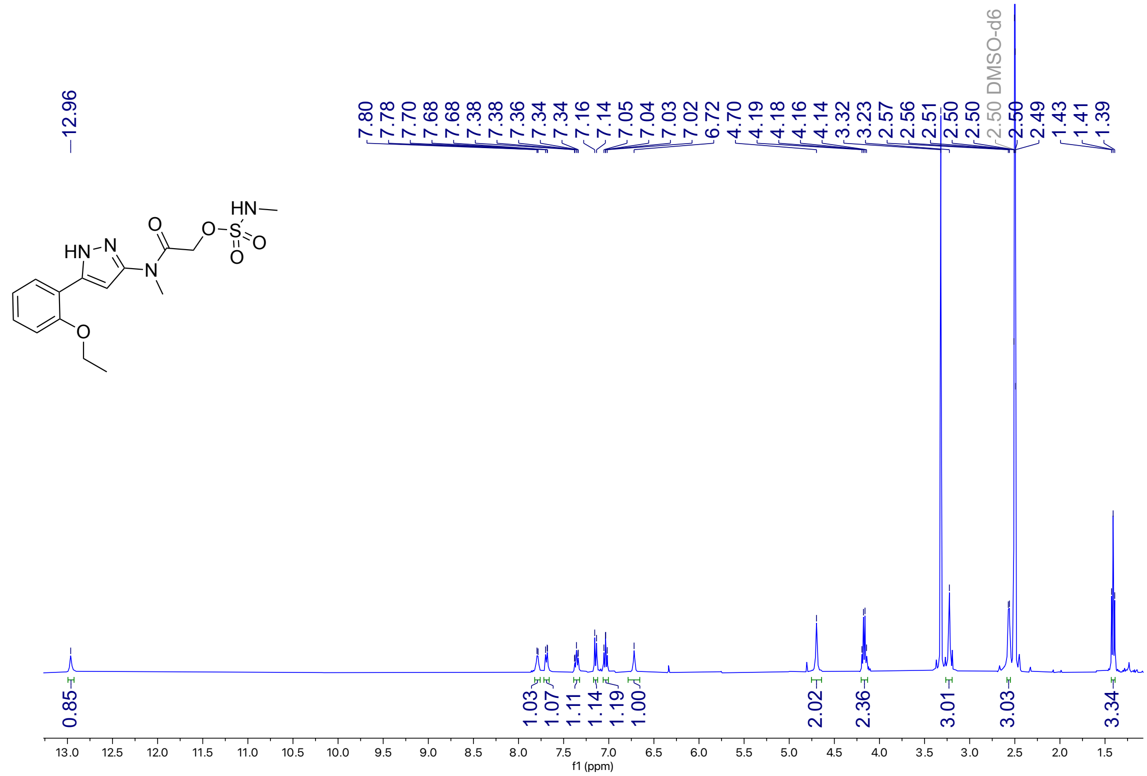

Figure S25: ^{13}C NMR (101 MHz, DMSO-d_6) for **6f**

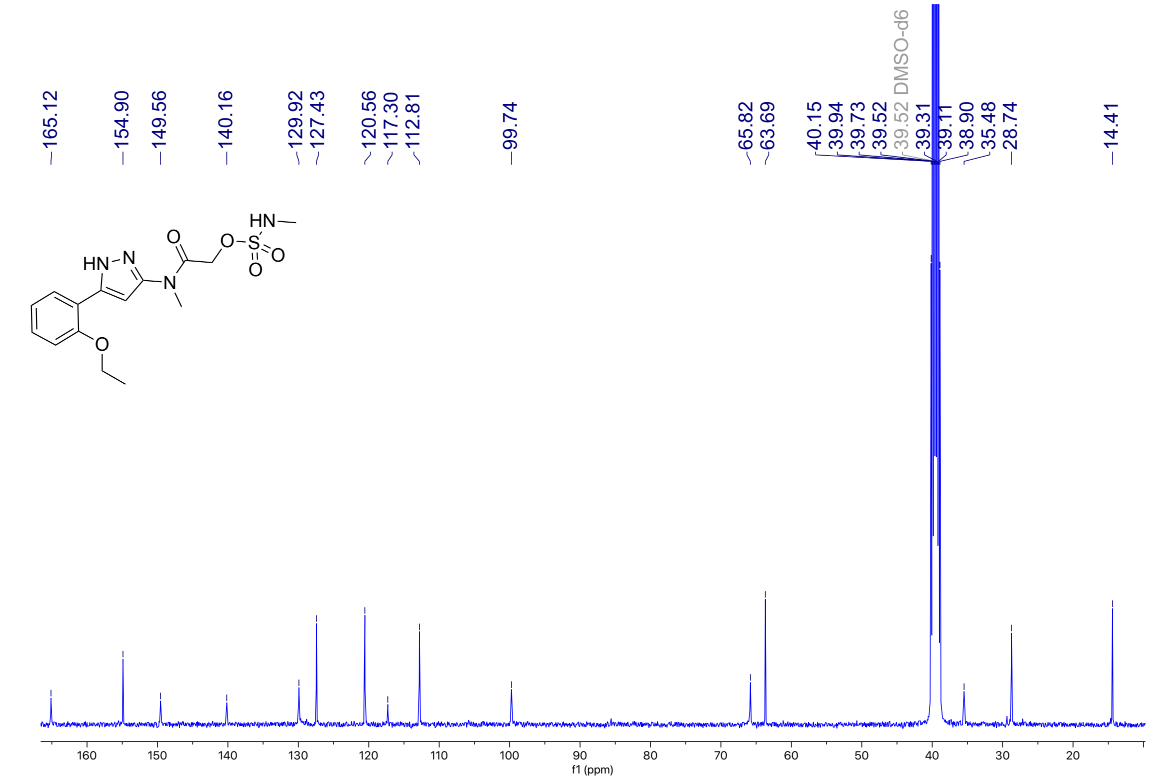

Figure S26: ^{1}H NMR (400 MHz, DMSO-d_6) for **7a**

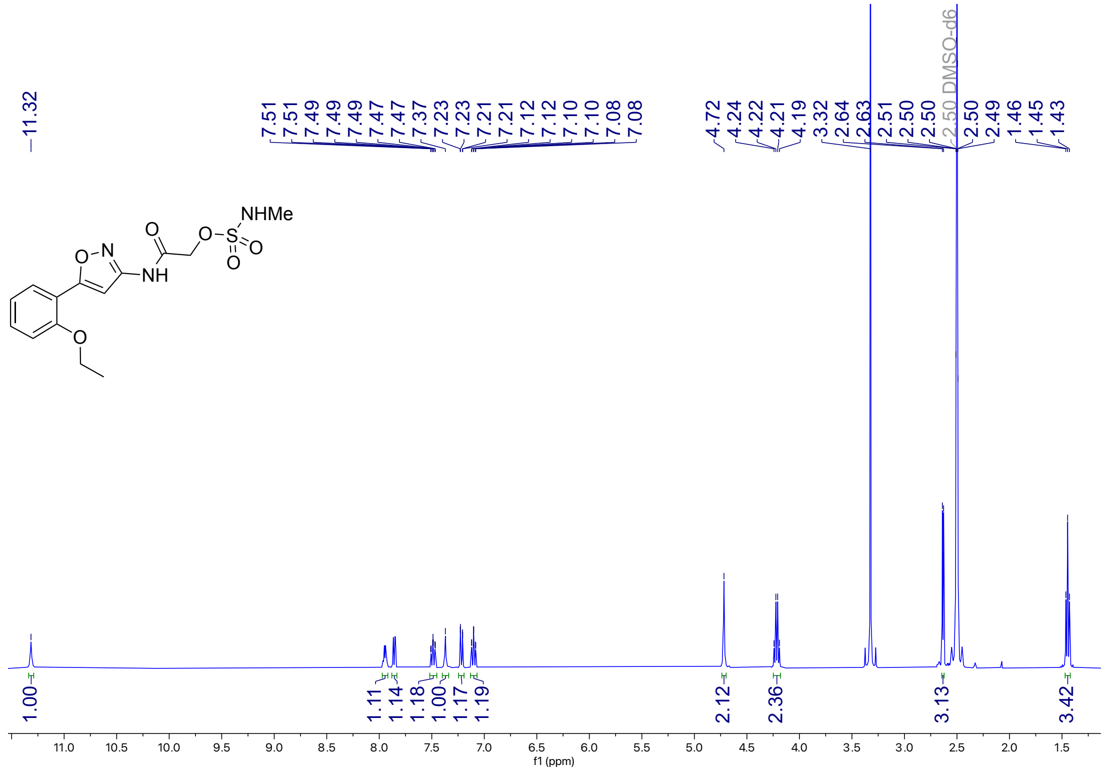

Figure S27: ^{13}C NMR (101 MHz, DMSO-d_6) for **7a**

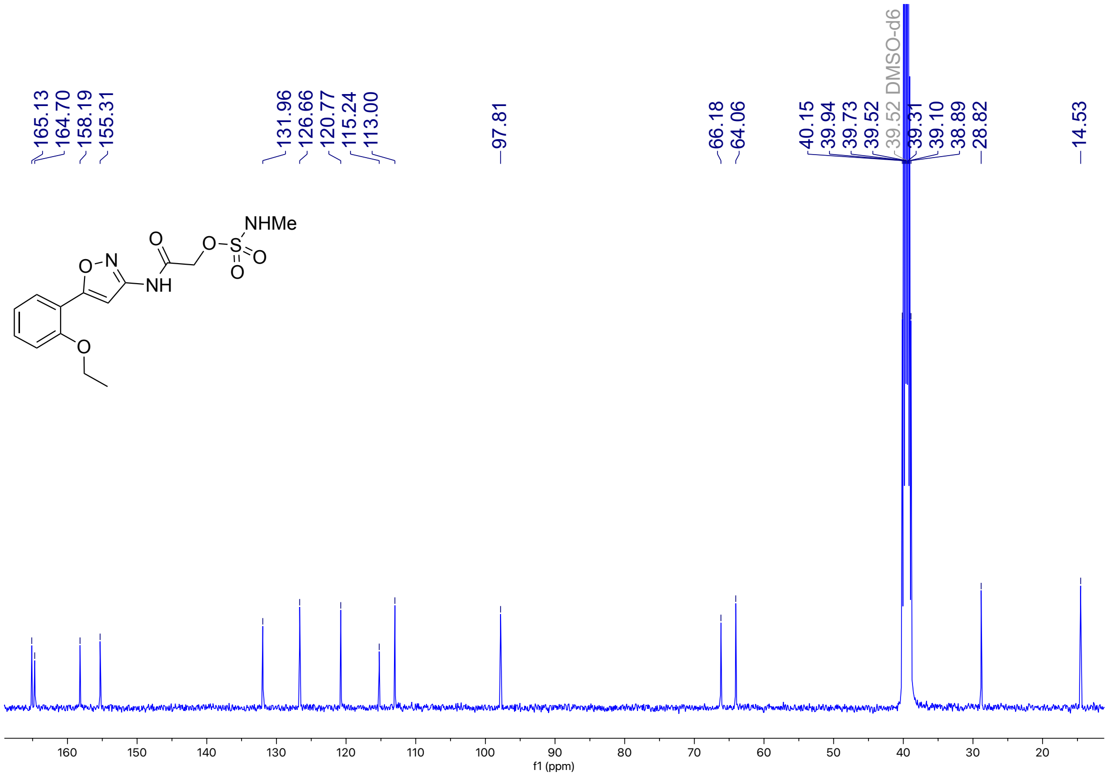

Figure S28: ^{1}H NMR (401 MHz, DMSO-d_6) for **7b**

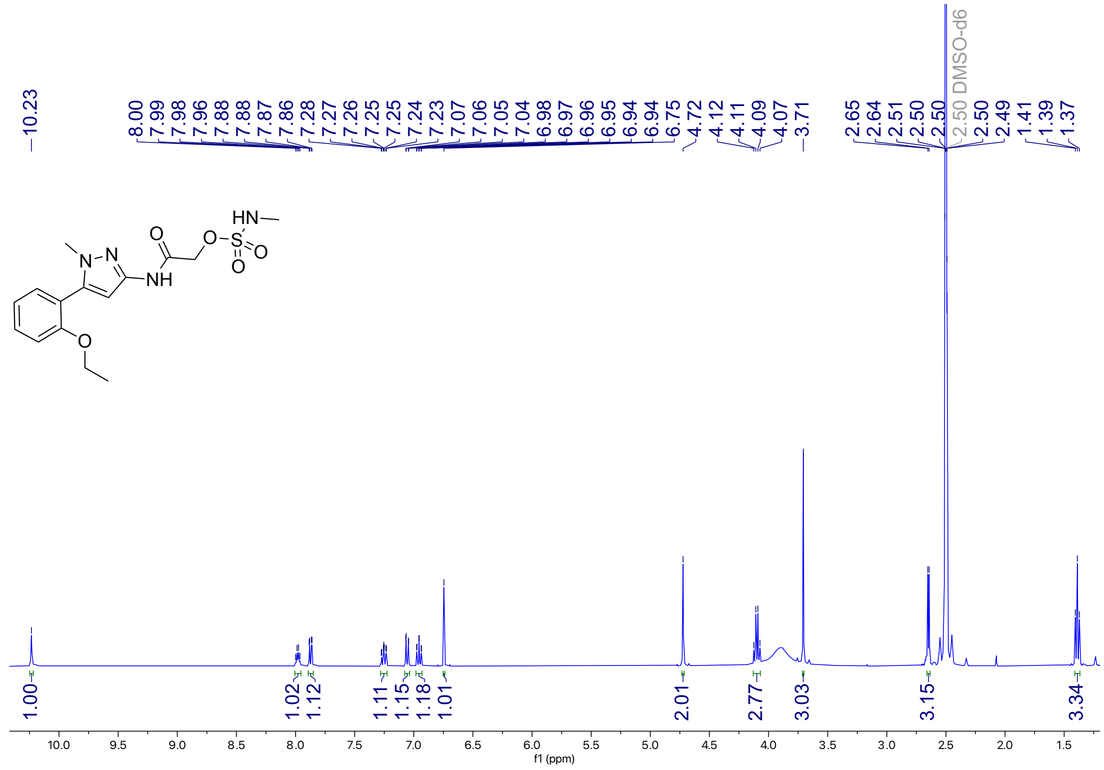

Figure S29: ^{13}C NMR (101 MHz, DMSO-d_6) for **7b**

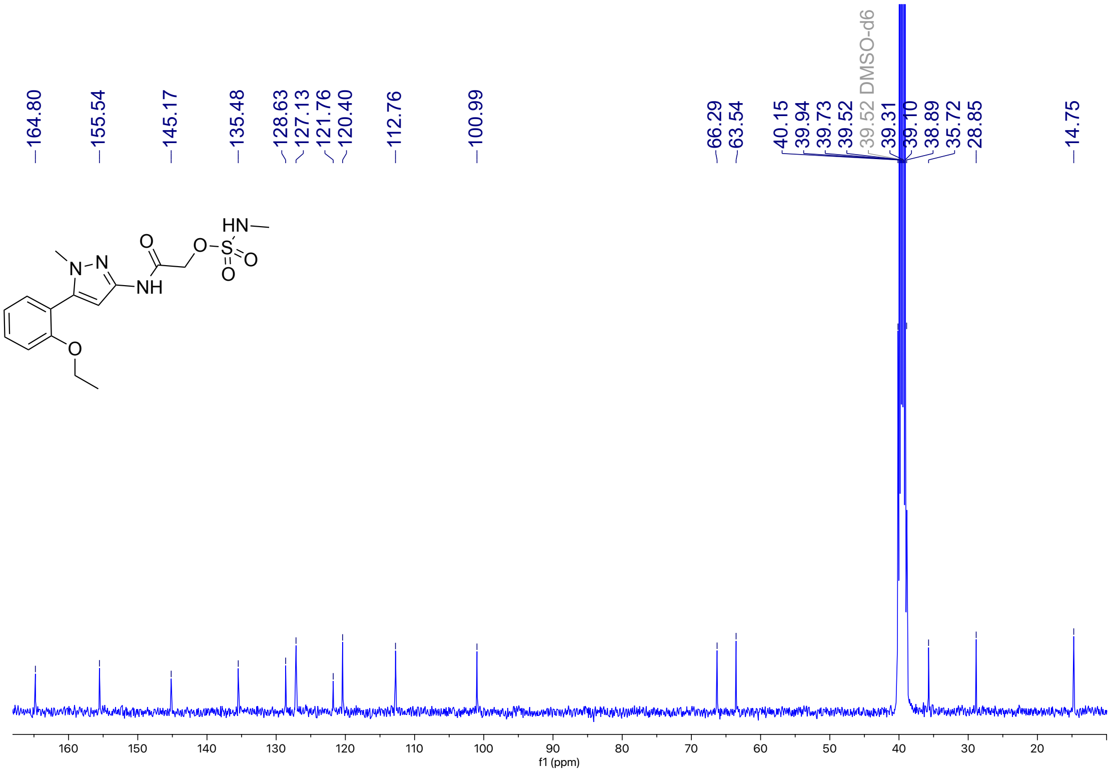

Figure S30: ^{1}H NMR (401 MHz, DMSO-d_6) for **7c**

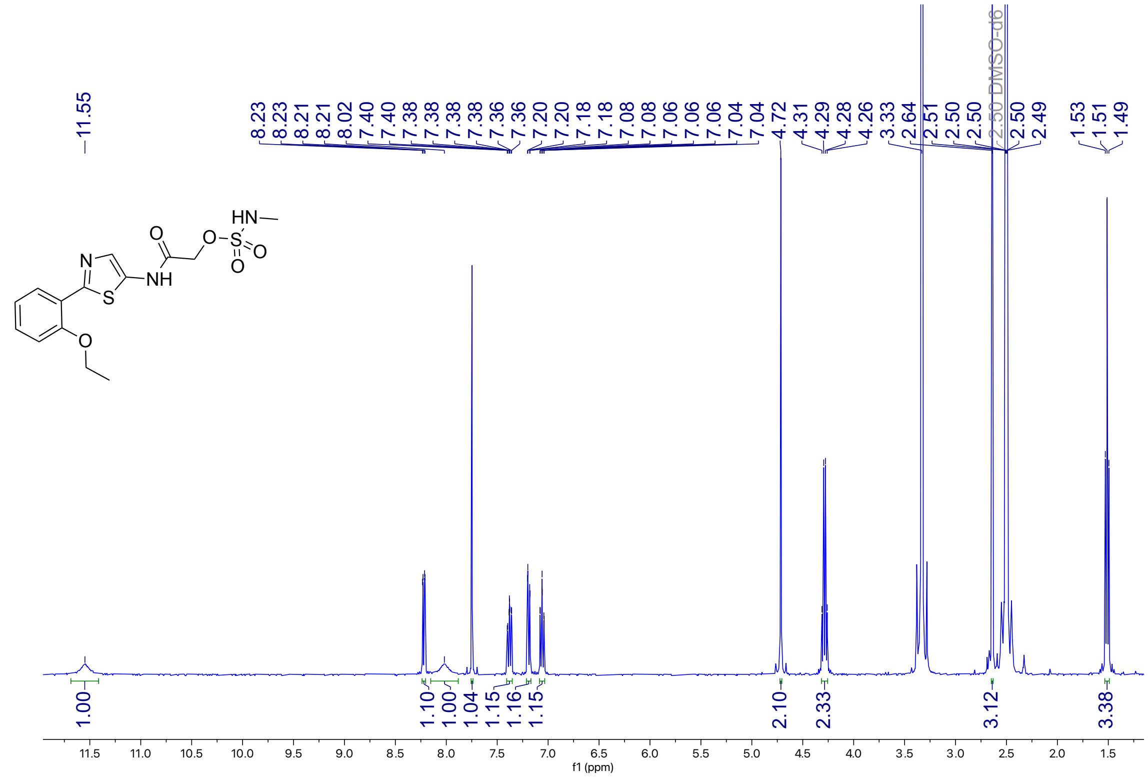

Figure S31: ^{13}C NMR (101 MHz, DMSO-d_6) for **7c**

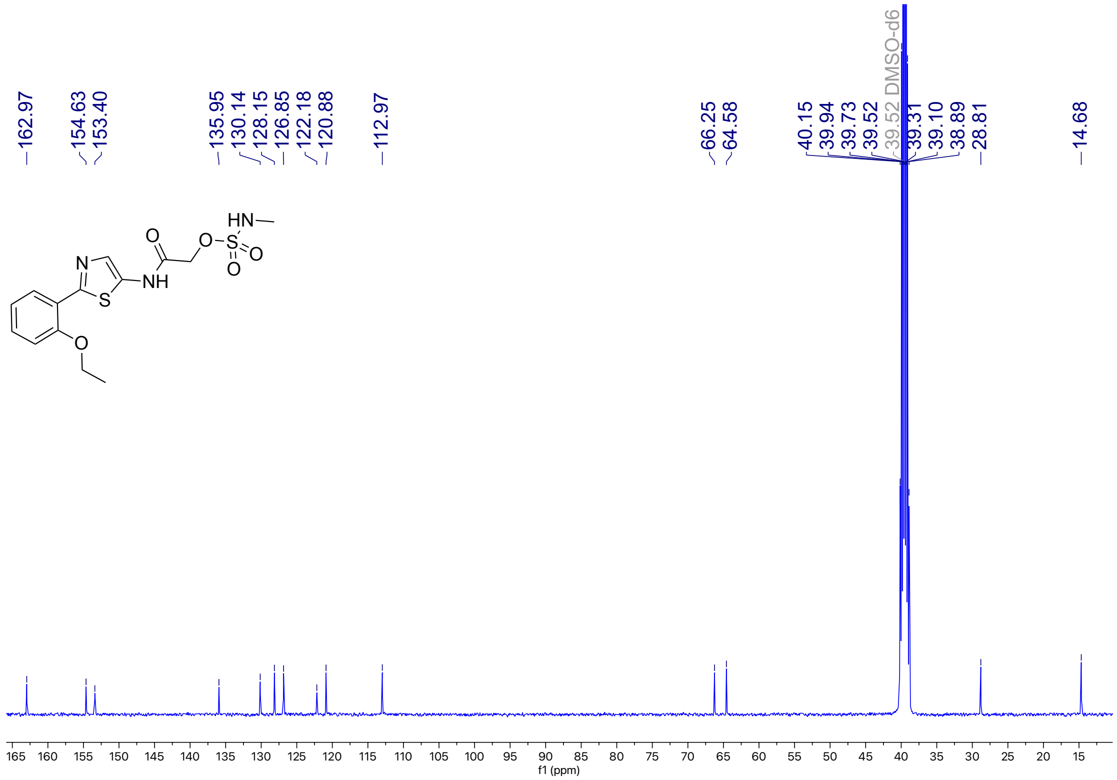

Figure S32: ^{1}H NMR (401 MHz, DMSO-d_6) for **7d**

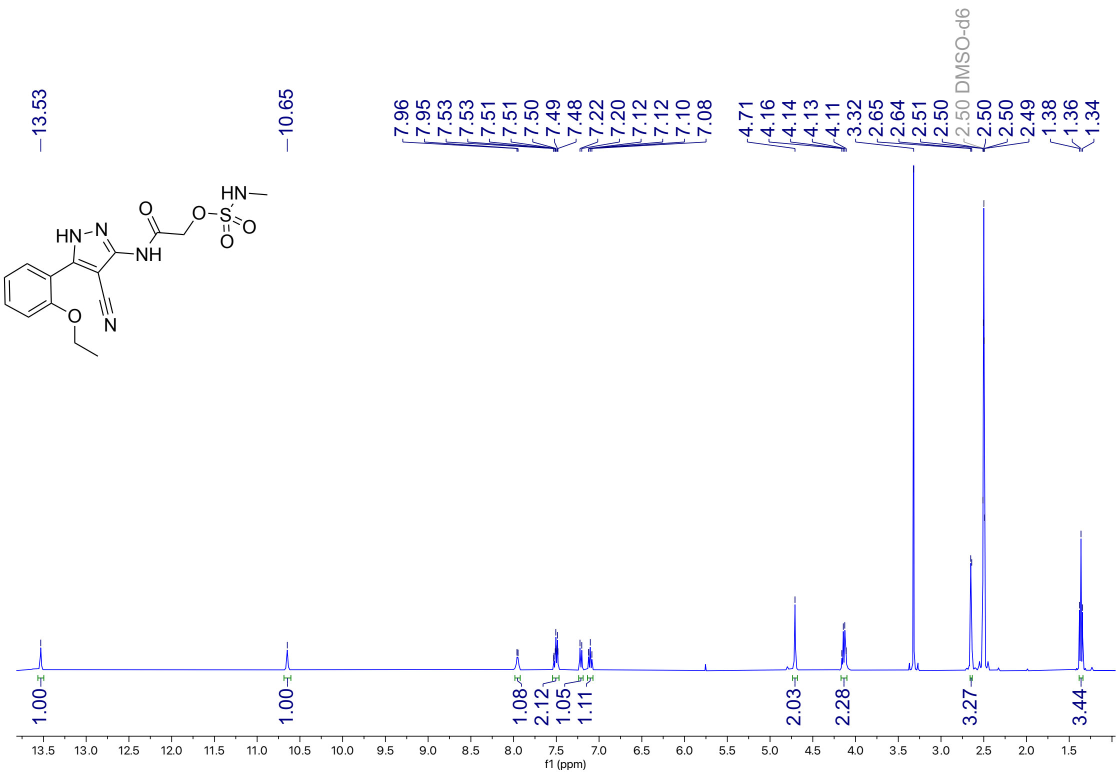

Figure S33: ^{13}C NMR (101 MHz, DMSO-d_6) for **7d**

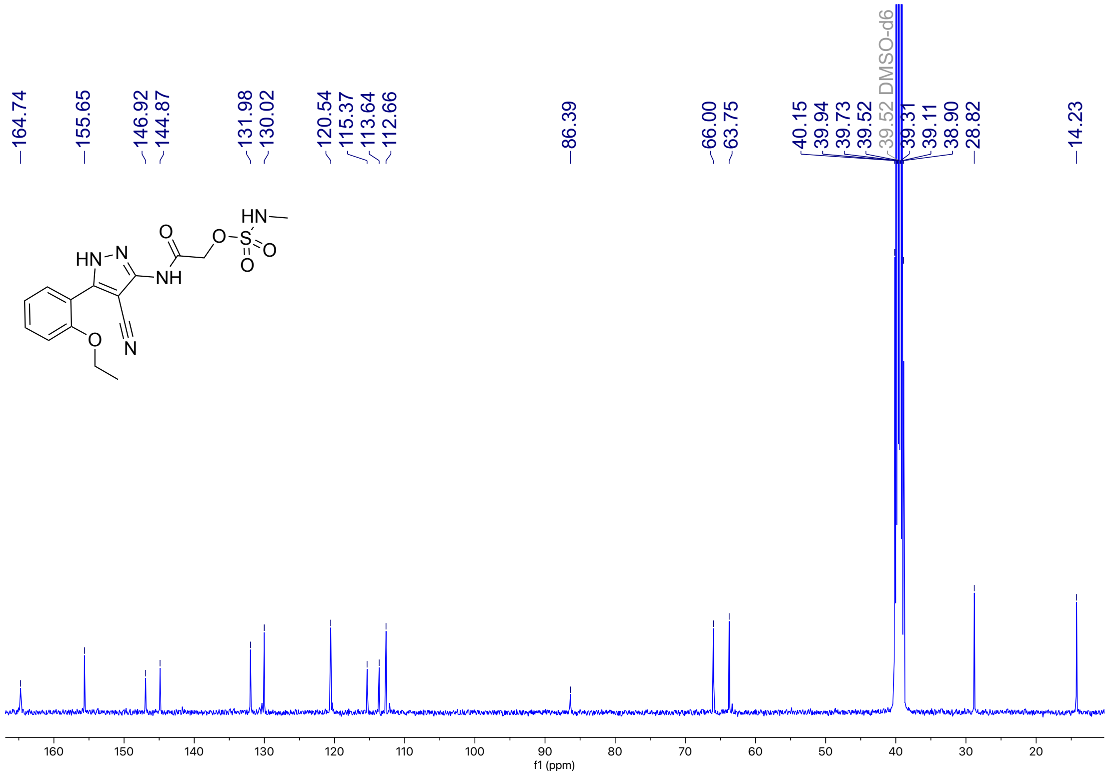

Figure S34: ^{1}H NMR (401 MHz, DMSO-d_6) for **8a**

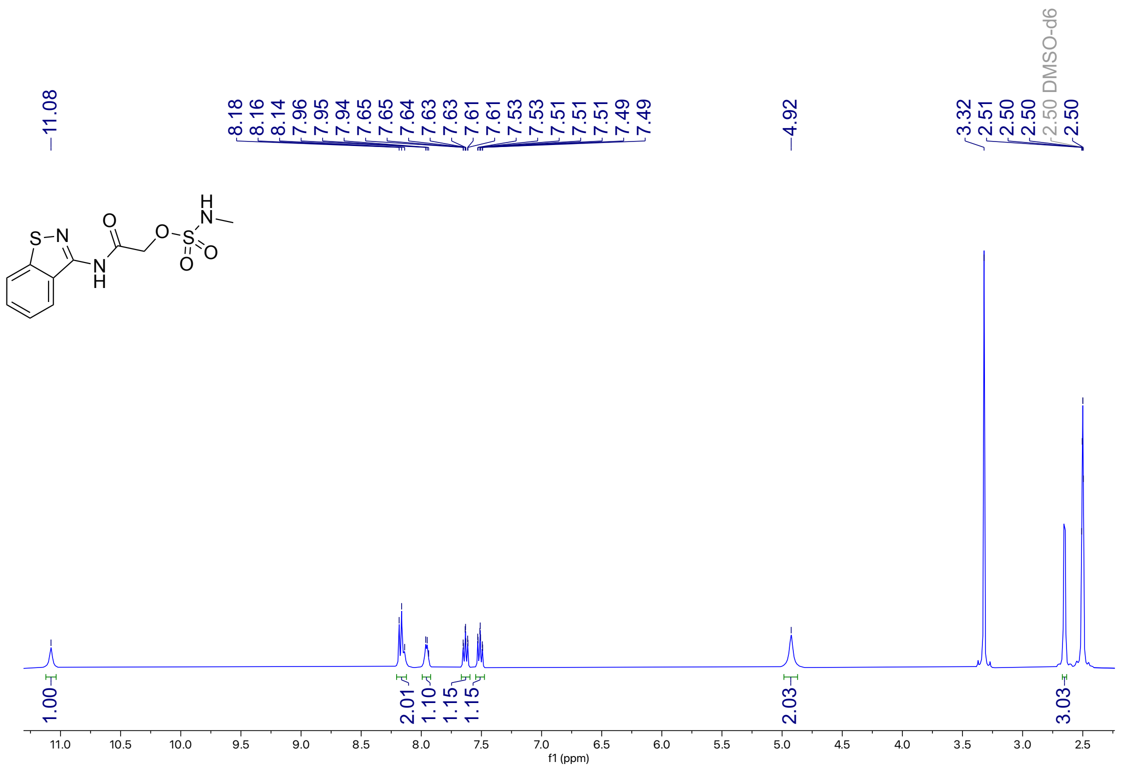

Figure S35: ^{13}C NMR (101 MHz, DMSO-d_6) for **8a**

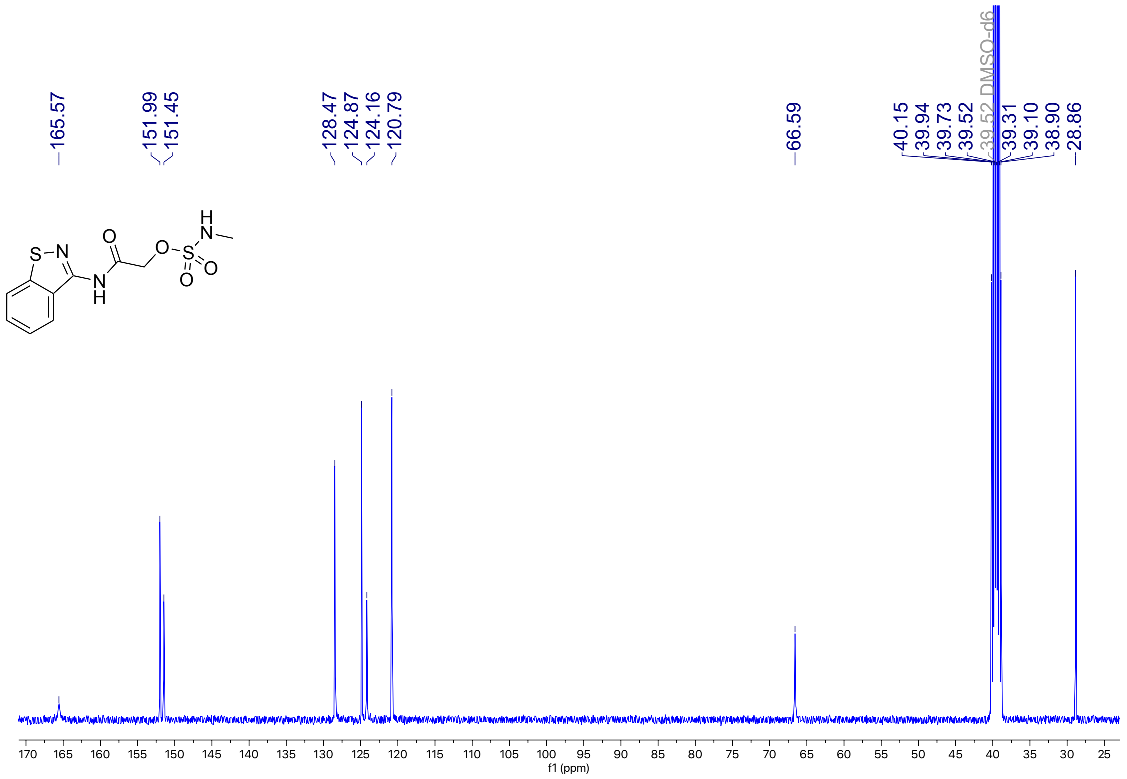

Figure S36: ^{1}H NMR (401 MHz, DMSO-d_6) for **8b**

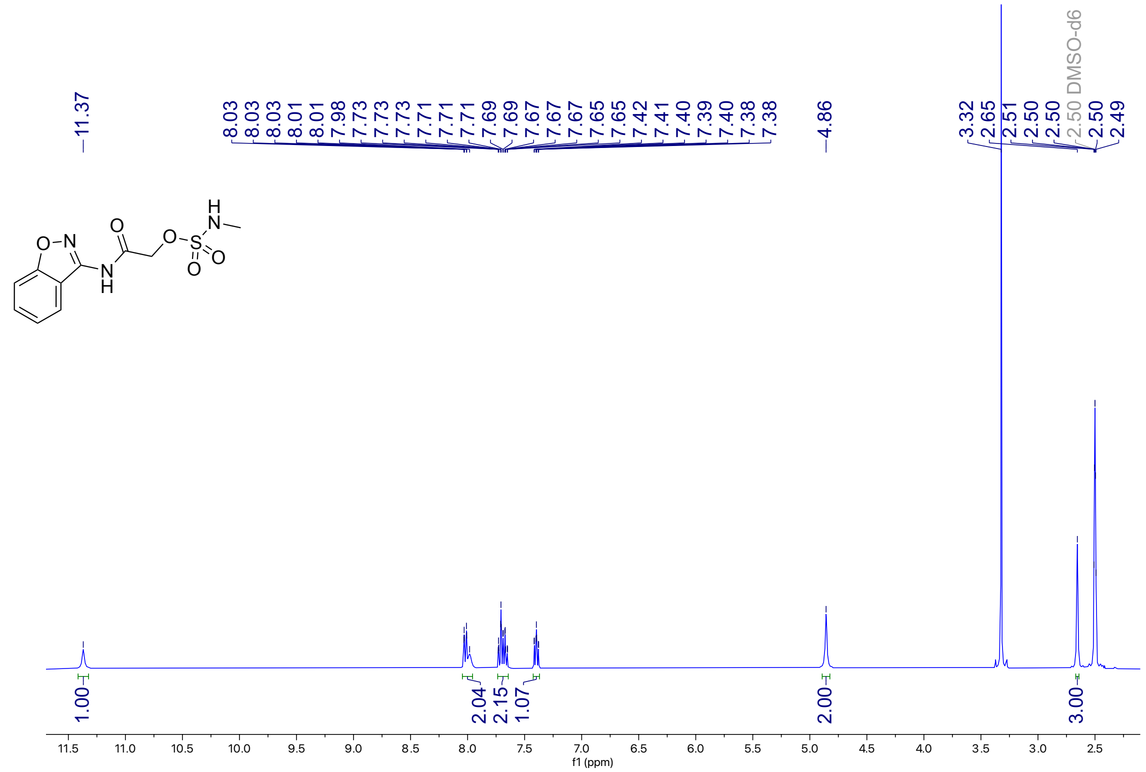

Figure S37: ^{13}C NMR (101 MHz, DMSO-d_6) for **8b**

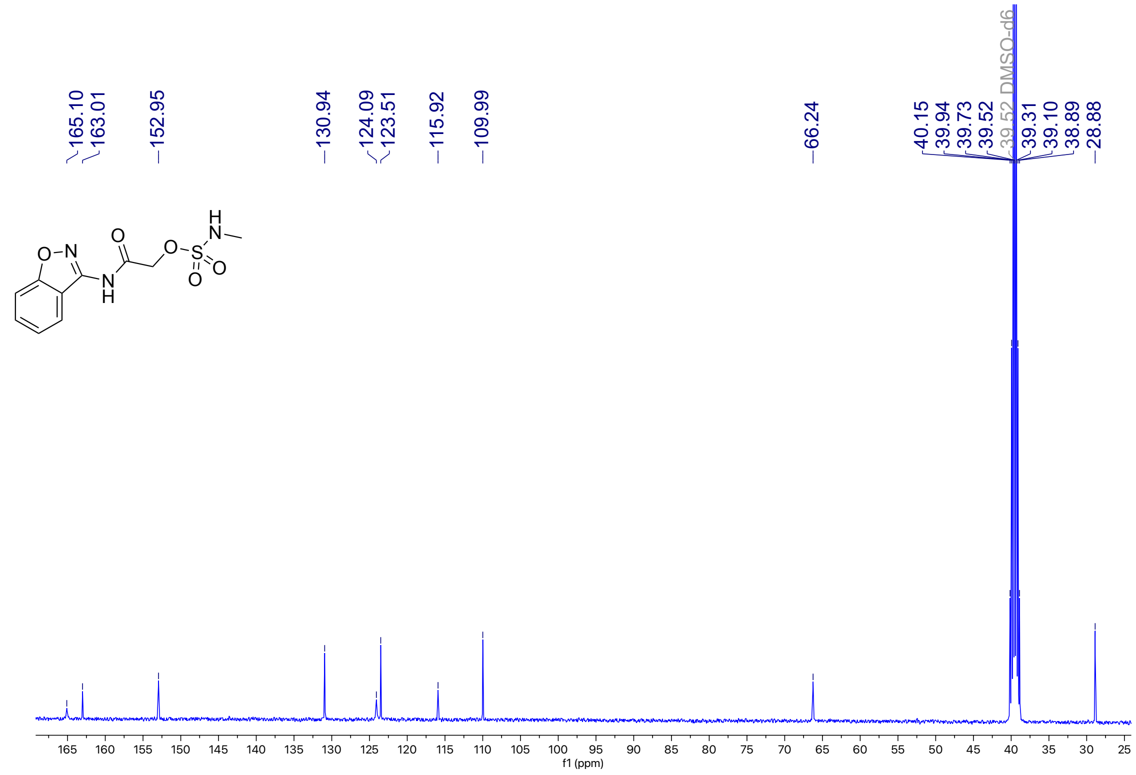

Figure S38: ^{1}H NMR (401 MHz, DMSO-d_6) for **8c**

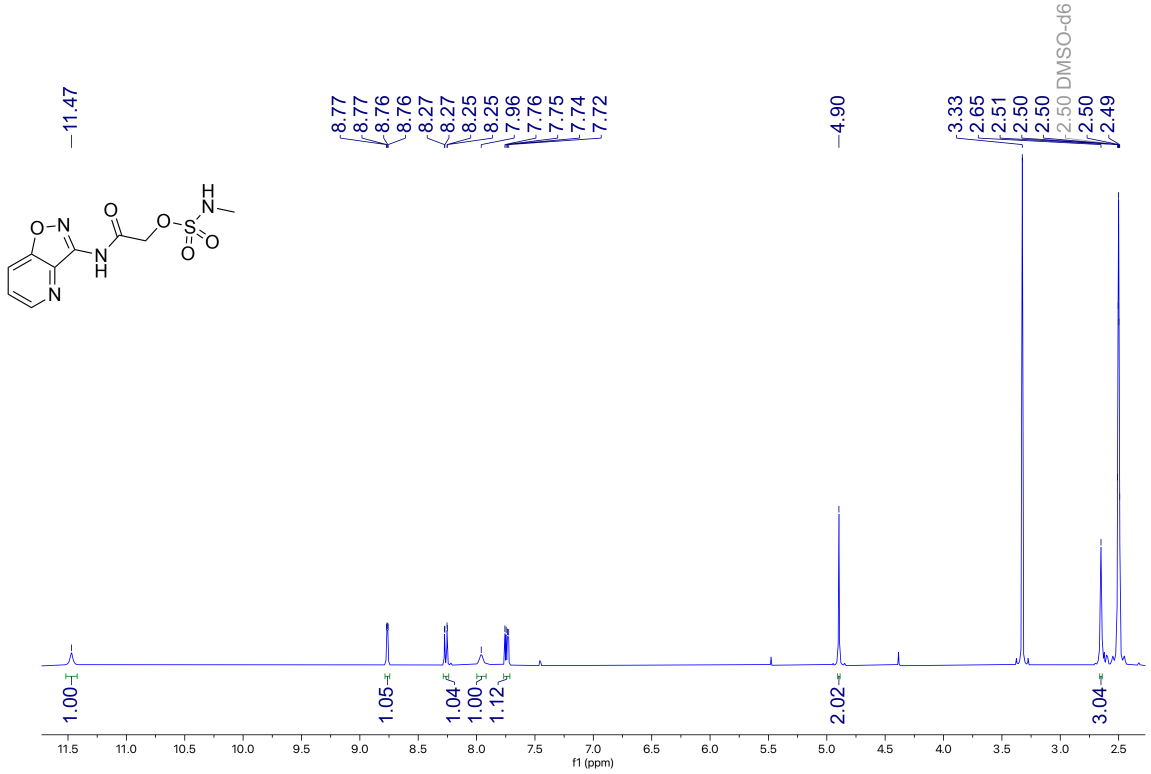

Figure S39: ^{13}C NMR (101 MHz, DMSO-d_6) for **8c**

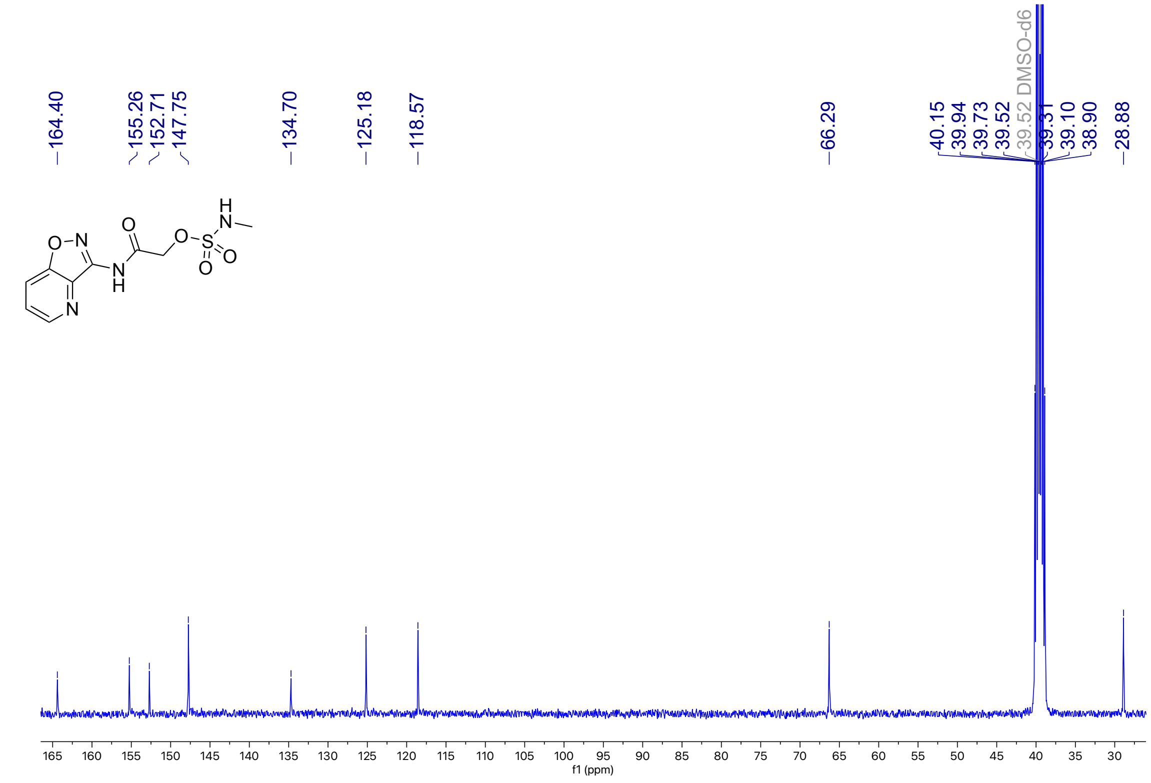

Figure S40: ^{1}H NMR (401 MHz, DMSO-d_6) for **9a**

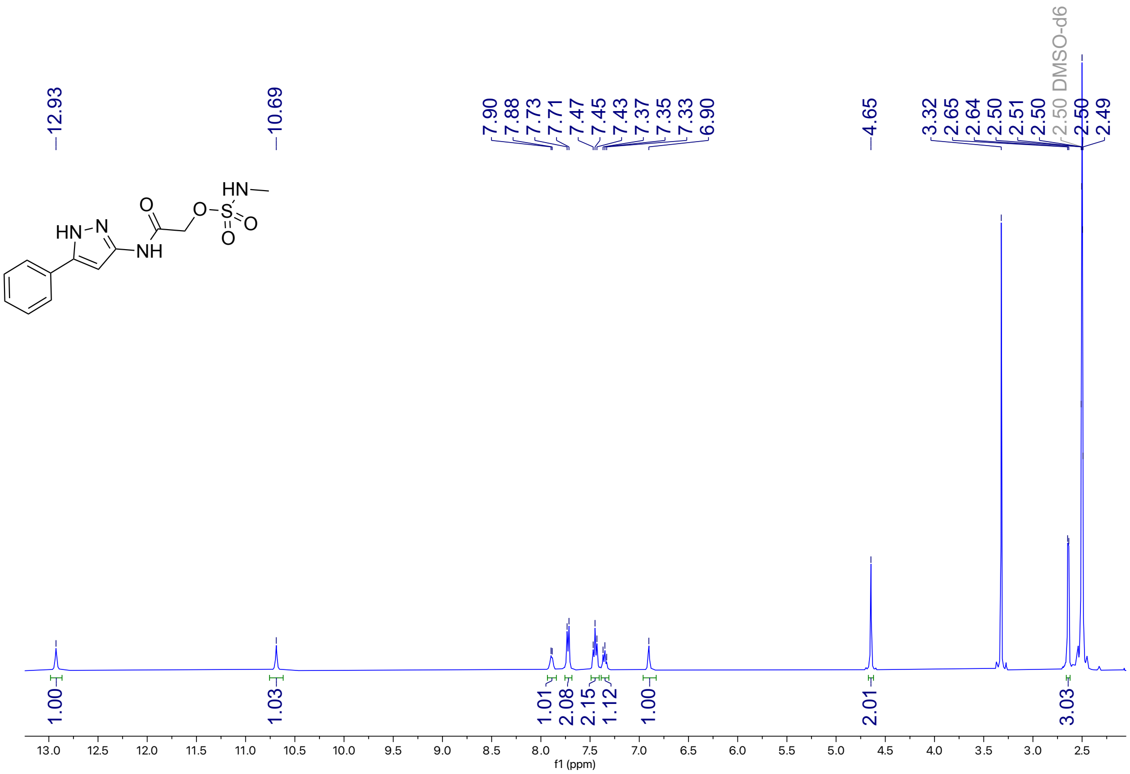

Figure S41: ^{13}C NMR (101 MHz, DMSO-d_6) for **9a**

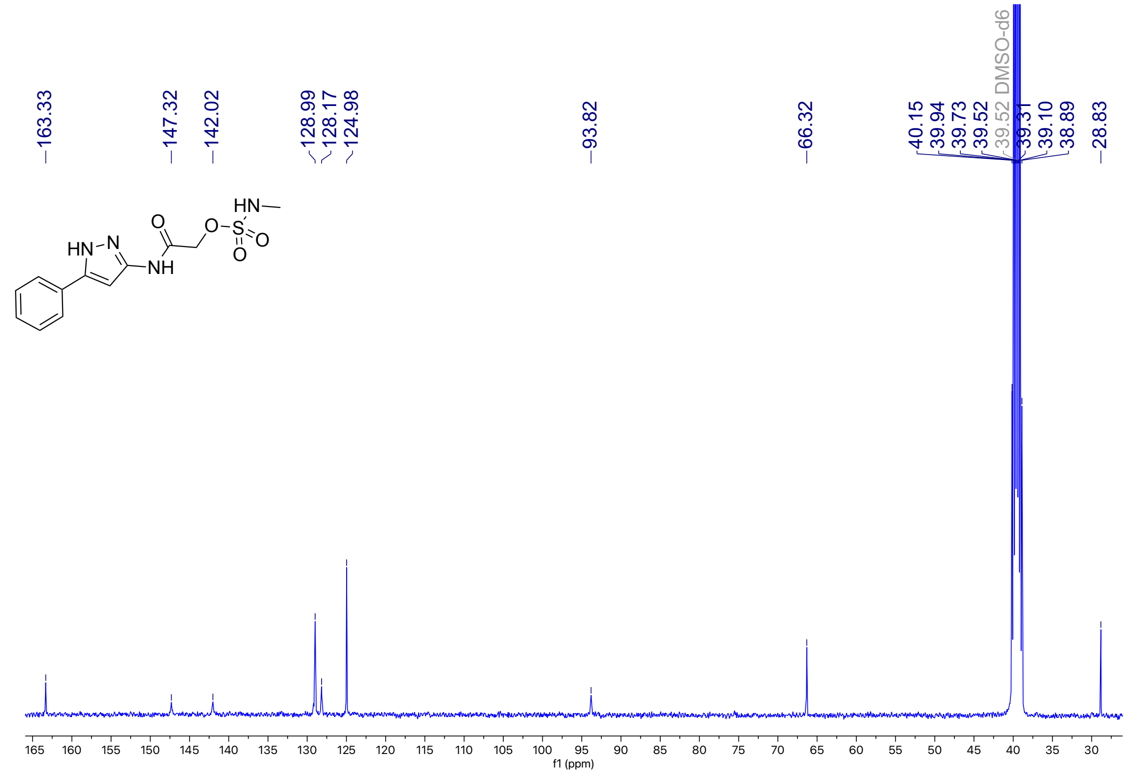

Figure S42: ^{1}H NMR (400 MHz, DMSO-d_6) for **9b**

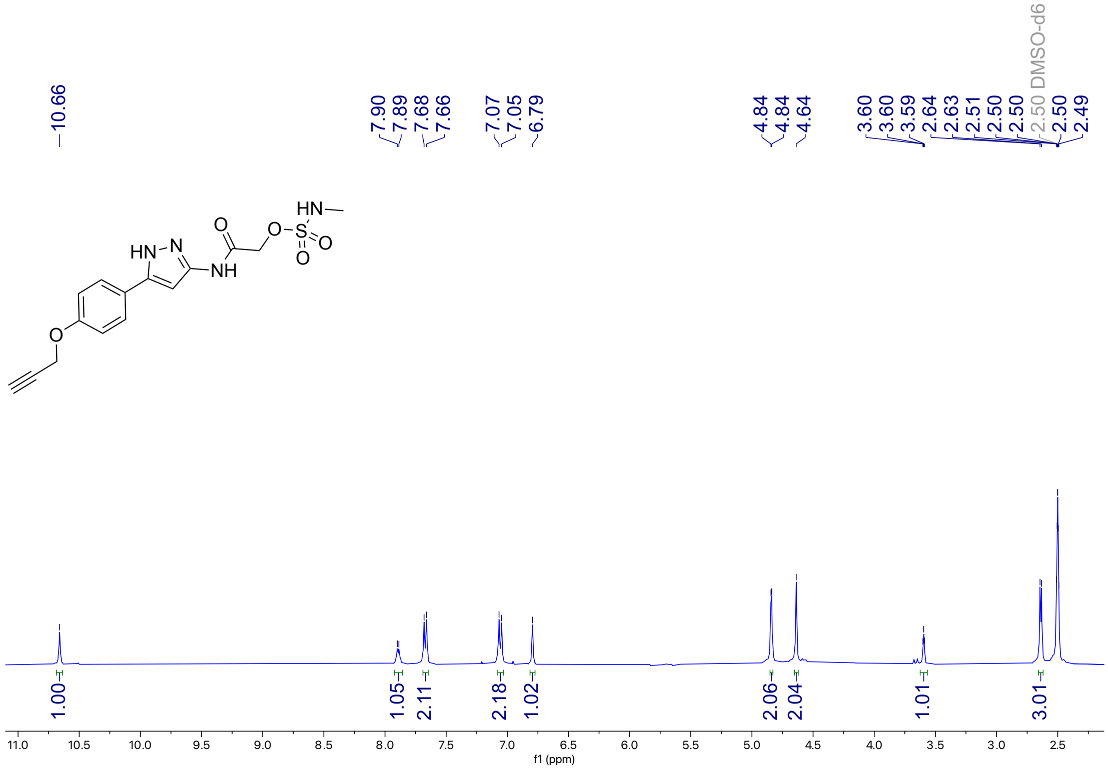

Figure S43: ^{13}C NMR (214 MHz, DMSO-d_6) for **9b**

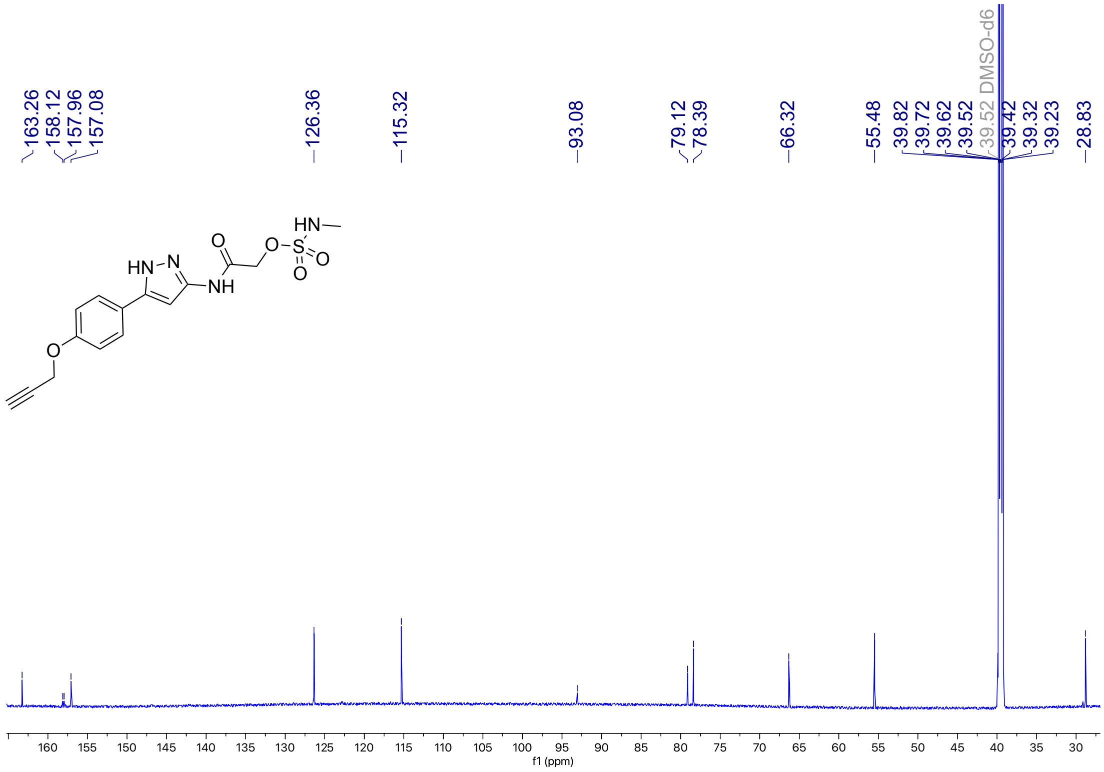

Figure S44: ^{1}H NMR (400 MHz, DMSO-d_6) for **9c**

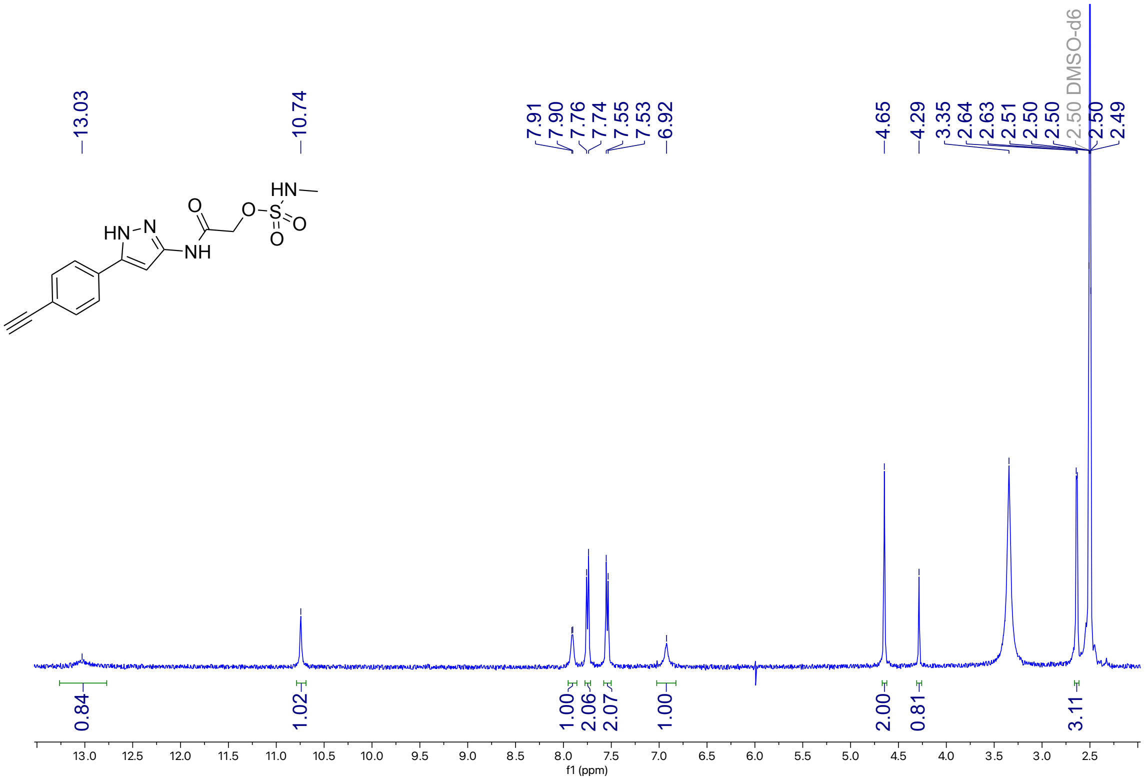

Figure S45: ^{13}C NMR (214 MHz, DMSO-d_6) for **9c**

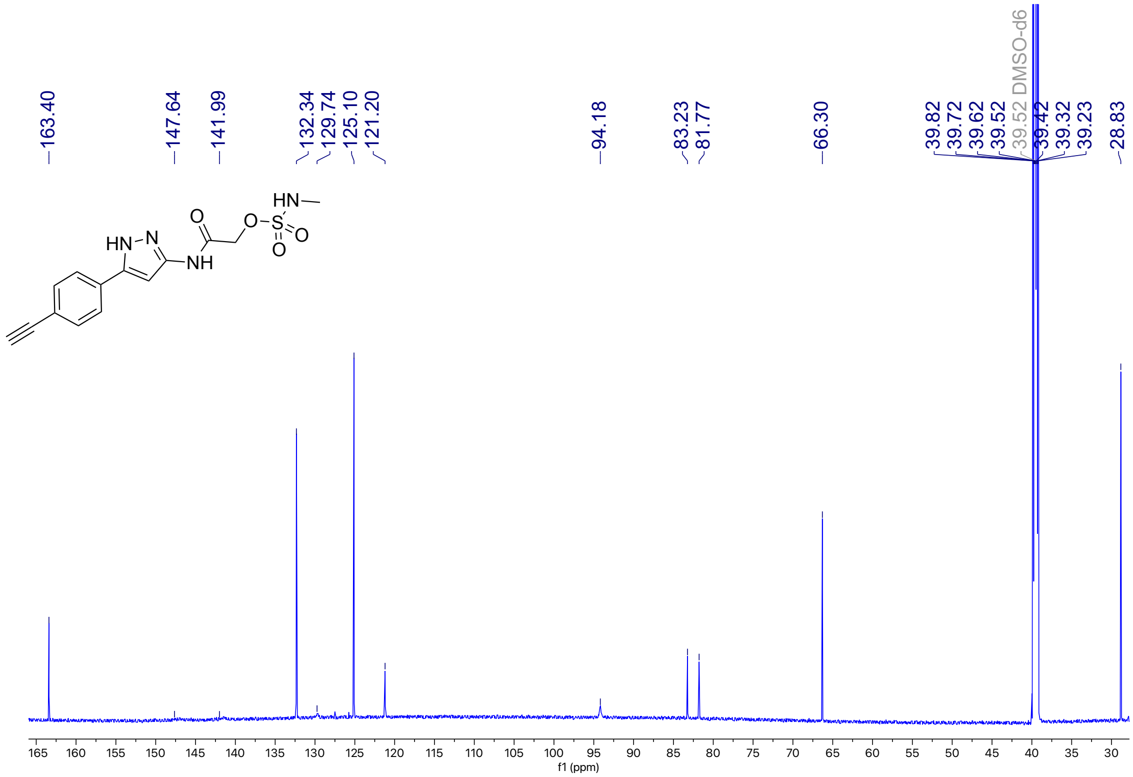

HPLC analyses

Figure S46. HPLC trace of **1**

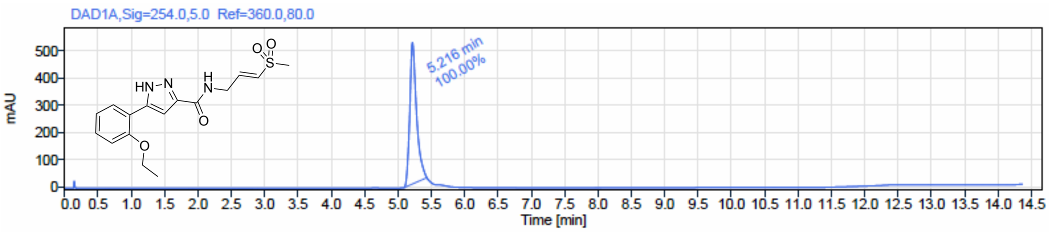

Signal Name DAD1A

RT (min)	Signal description	m/z	Purity UV(‰) / MS(%)	Area	Area%
5.216	DAD1A,Sig=254.0,5.0 Ref=360.0,80.0			3652.8	100.00

Figure S47. HPLC trace of **2**

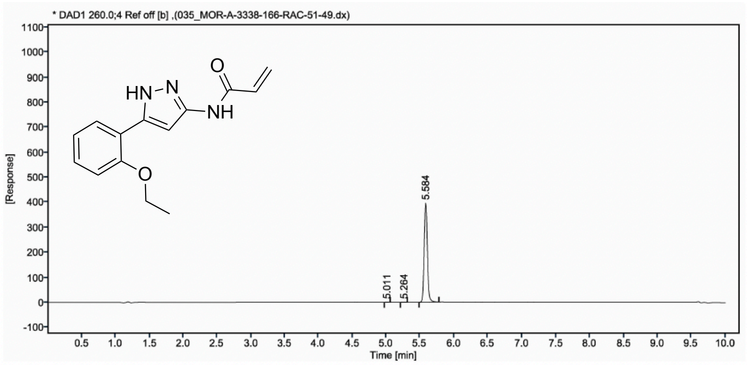

Sr.No.	RT	Area	Height	% Area
1	5.01	2	1	0.16
2	5.26	2	1	0.15
3	5.58	1286	395	99.69

Figure S48. HPLC trace of **3**

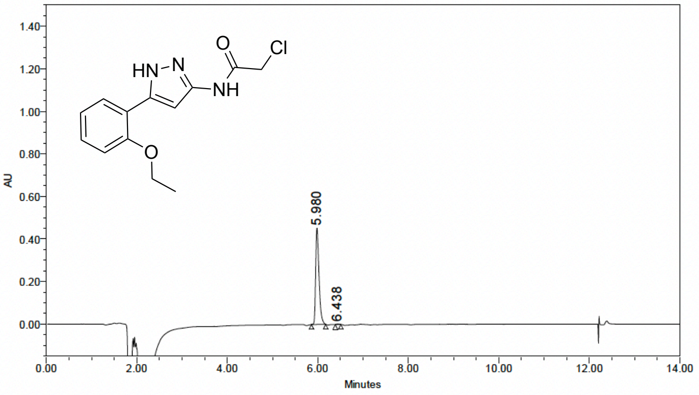

Peak Results

	Name	RT	Height (μV)	Area (μV*sec)	% Area
1		5.980	452570	2320222	99.49
2		6.438	2795	11856	0.51

Figure S49. HPLC trace of **4**

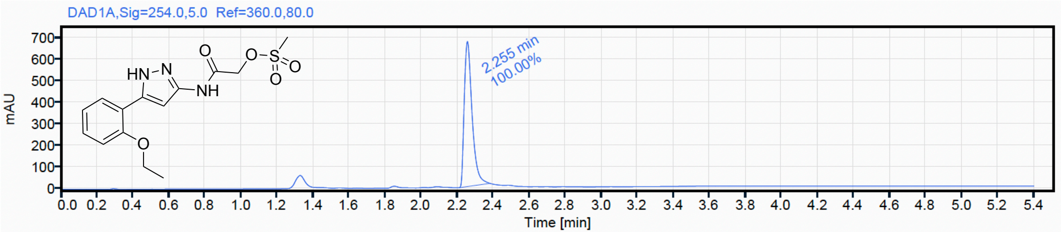

Signal Name	DAD1A		
**RT (min)**	**Signal description**	**Area**	**Area%**
2.255	DAD1A,Sig=254.0,5.0 Ref=360.0,80.0	2044.2	100.00

Figure S50. HPLC trace of **5**

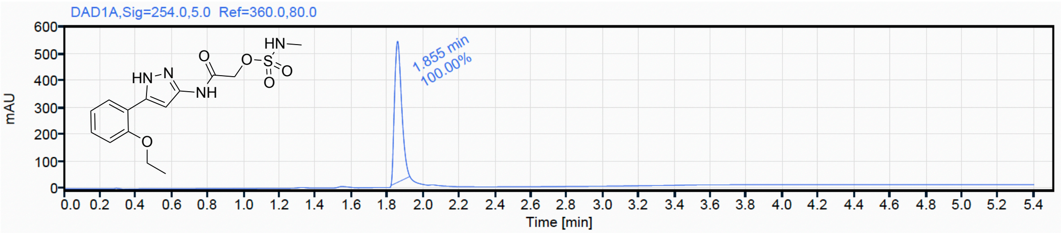

Signal Name	DAD1A		
**RT (min)**	**Signal description**	**Area**	**Area%**
1.855	DAD1A,Sig=254.0,5.0 Ref=360.0,80.0	1446.9	100.00

Figure S51. HPLC trace of **6a**

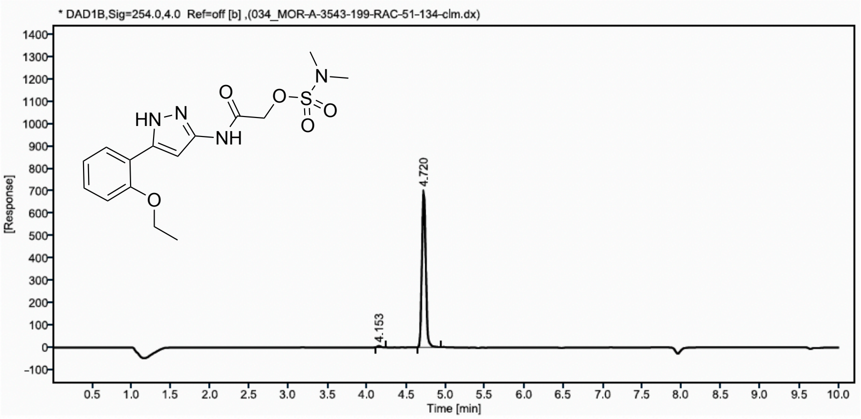

Sr.No.	RT	Area	Height	% Area
1	4,15	18	6	0,76
2	4,72	2404	704	99,24

Figure S52. HPLC trace of **6b**

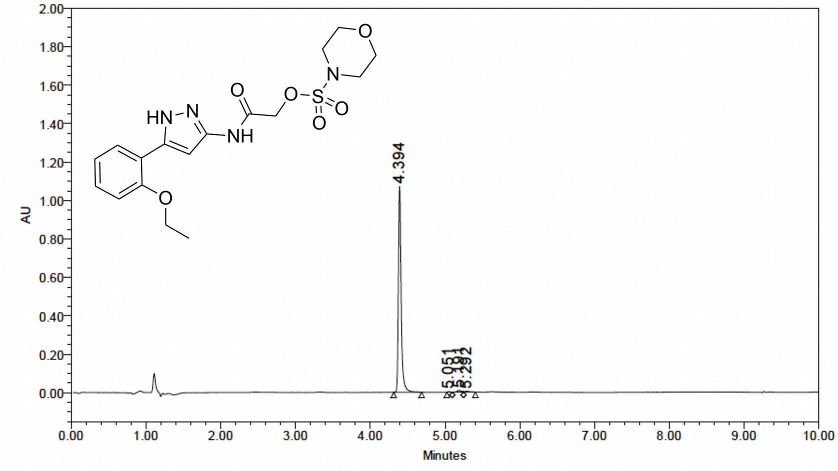

Peak Results

	Name	RT	Height (μV)	Area (μV*sec)	% Area
1		4.394	1072091	2779288	97.84
2		5.051	2777	5985	0.21
3		5.191	7935	33688	1.19
4		5.292	4552	21689	0.76

Figure S53. HPLC trace of **6c**

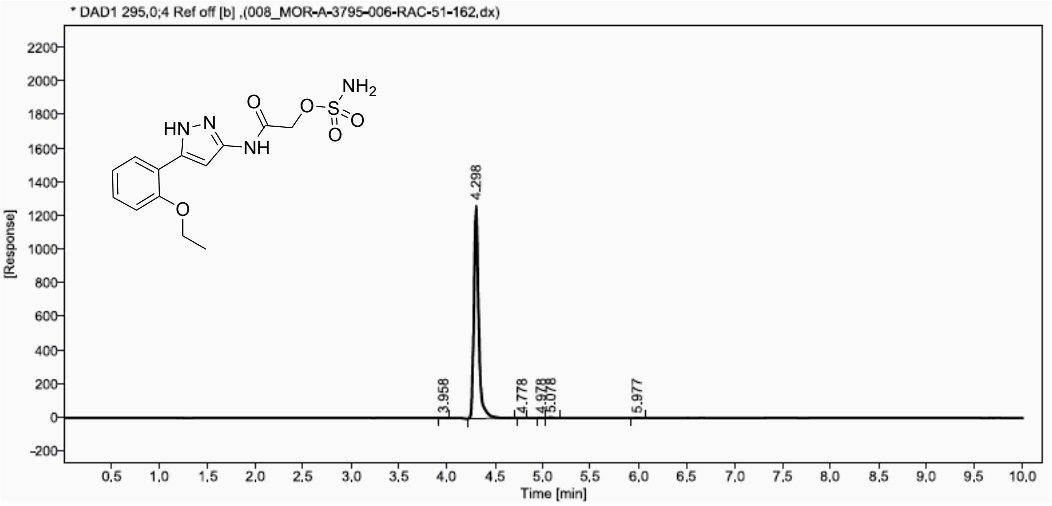

Sr.No.	RT	Area	Height	% Area
1	3,96	3	1	0,07
2	4,30	4445	1275	99,38
3	4,78	1	0	0.03
4	4,98	4	1	0,09
5	5,08	15	4	0.33
6	5,98	4	1	0,10

Figure S54. HPLC trace of **6d**

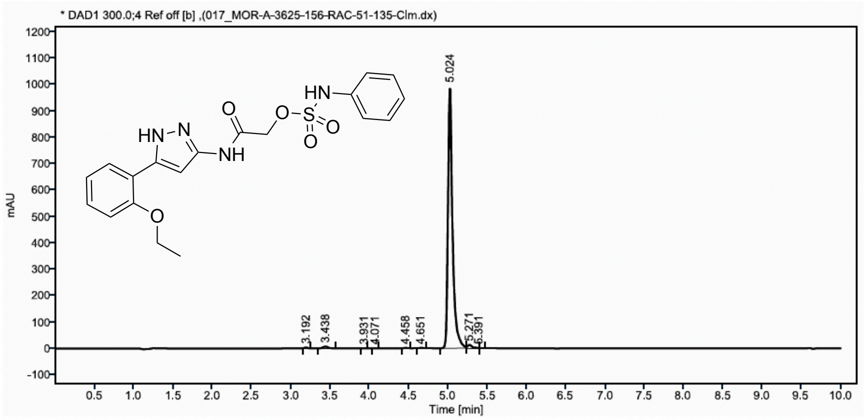

Sr.No.	RT	Area	Height	% Area
1	3.19	9	3	0.23
2	3.44	29	7	0.71
3	3.93	1	0	0.03
4	4.07	1	0	0.02
5	4.46	4	1	0.10
6	4.65	3	1	0.08
7	5.02	4004	993	97.56
8	5.27	51	13	1.25
9	5.39	1	0	0.02

Figure S55. HPLC trace of **6e**

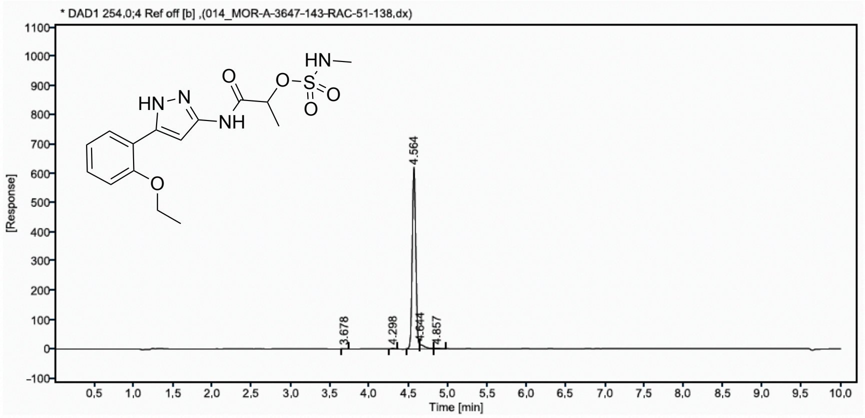

Sr.No.	RT	Area	Height	% Area
1	3.68	1	0	0.06
2	4.30	6	2	0.28
3	4.56	1966	617	96.23
4	4.64	61	14	2.97
5	4.86	9	2	0.46

Figure S56. HPLC trace of **6f**

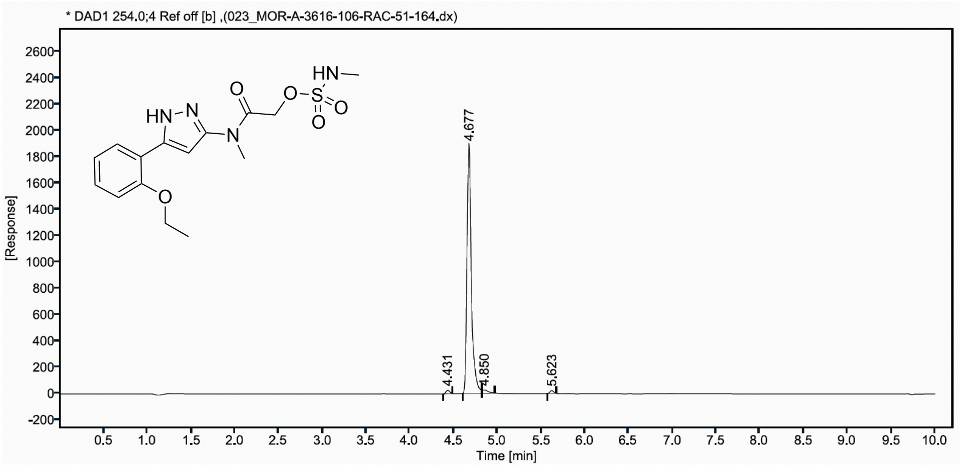

Sr.No.	RT	Area	Height	% Area
1	4.43	75	26	1.08
2	4.68	6649	1883	96.41
3	4.85	107	25	1.55
4	5.62	66	22	0.95

Figure S57. HPLC trace of **7a**

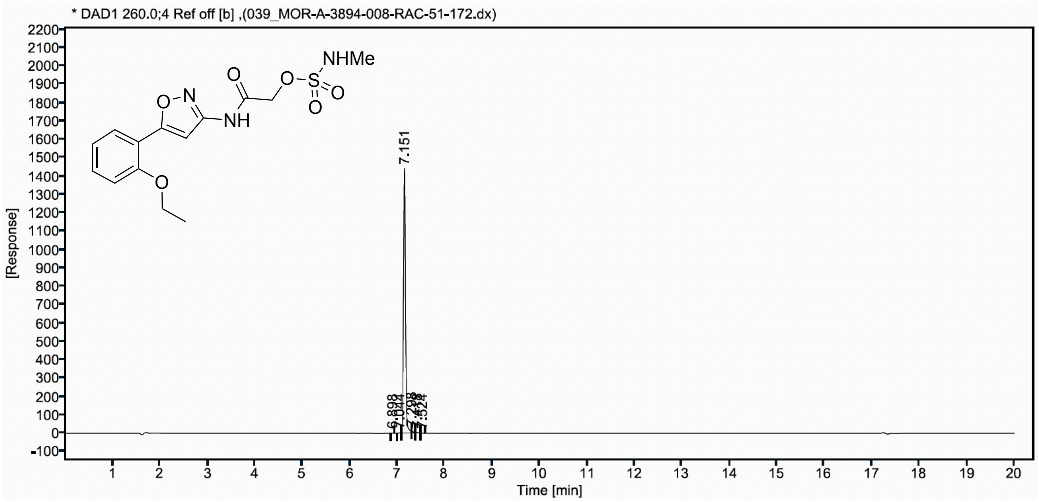

Sr.No.	RT	Area	Height	% Area
1	6.90	3	1	0.07
2	7.04	10	4	0.23
3	7.15	4320	1439	98.26
4	7.30	35	11	0.79
5	7.42	21	4	0.49
6	7.52	7	2	0.16

Figure S58. HPLC trace of **7b**

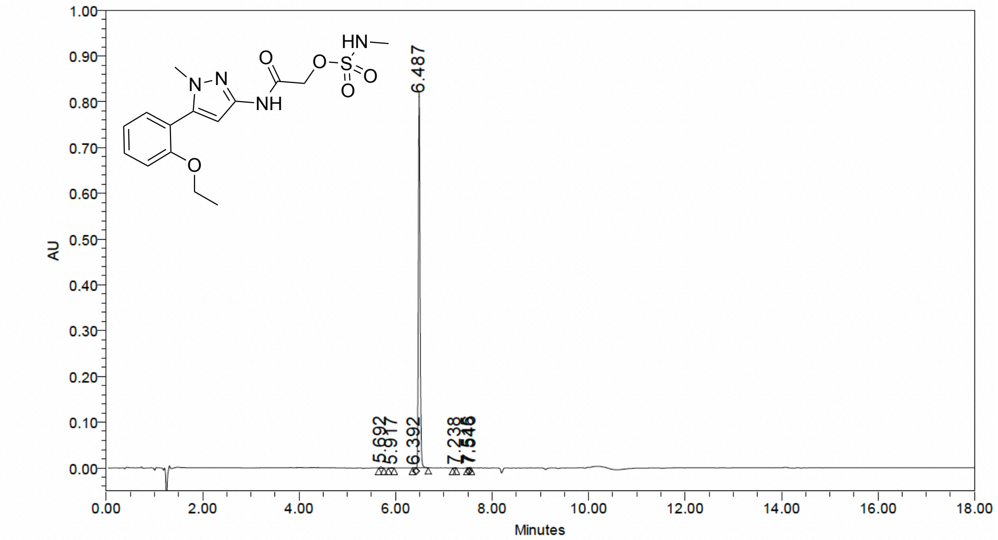

Peak Results

	RT	Area (µV*sec)	Height (µV)	% Area
1	5.692	5958	2519	0.29
2	5.917	1347	579	0.07
3	6.392	1426	658	0.07
4	6.487	2018110	820961	99.35
5	7.238	1630	1009	0.08
6	7.515	772	524	0.04
7	7.546	2007	1096	0.10

Figure S59. HPLC trace of **7c**

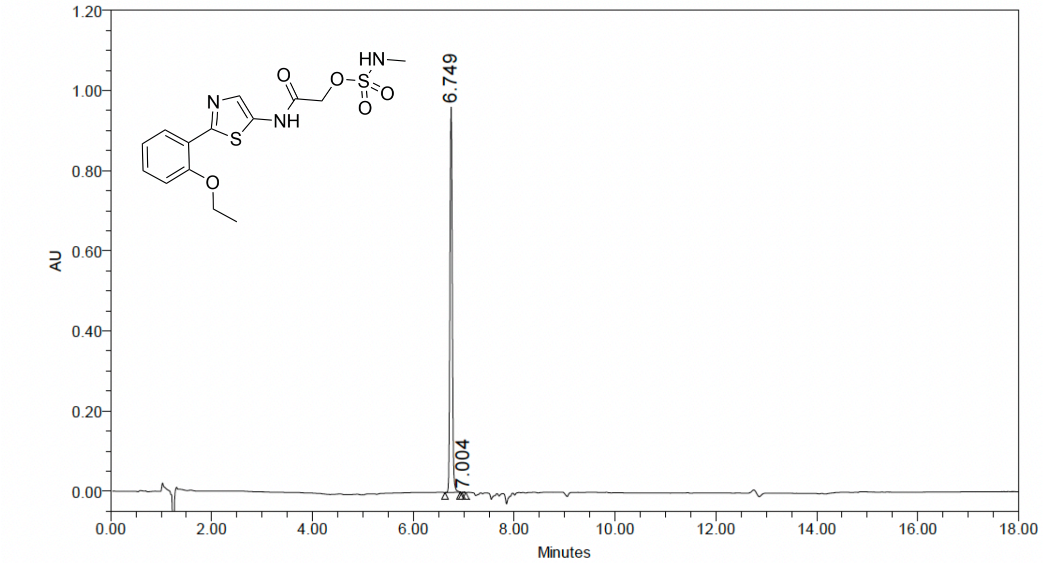

Peak Results

	RT	Area (µV*sec)	Height (µV)	% Area
1	6.749	3132122	962202	99.94
2	7.004	2011	751	0.06

Figure S60. HPLC trace of **7d**

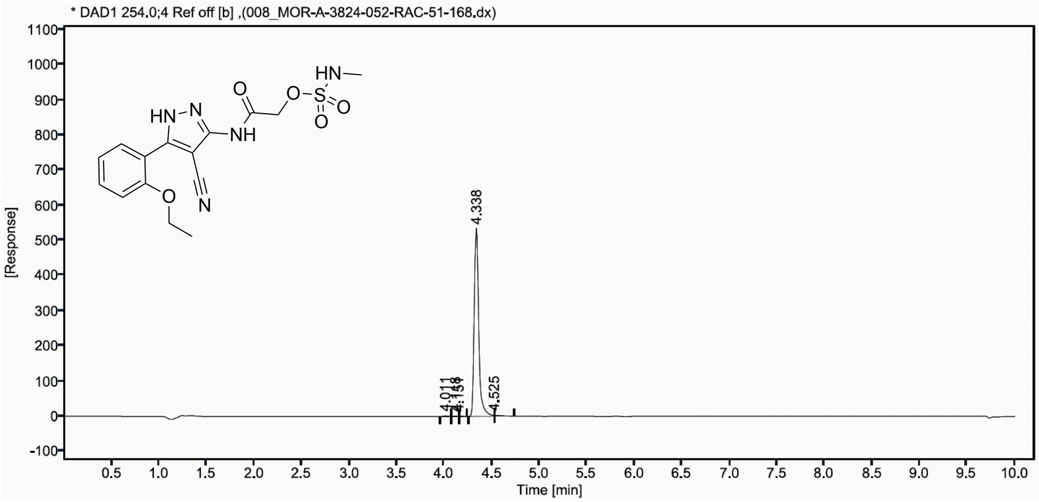

Sr.No.	RT	Area	Height	% Area
1	4.01	5	2	0.30
2	4.12	3	1	0.18
3	4.15	2	1	0.09
4	4.34	1775	533	98.60
5	4.52	15	3	0.83

Figure S61. HPLC trace of **8a**

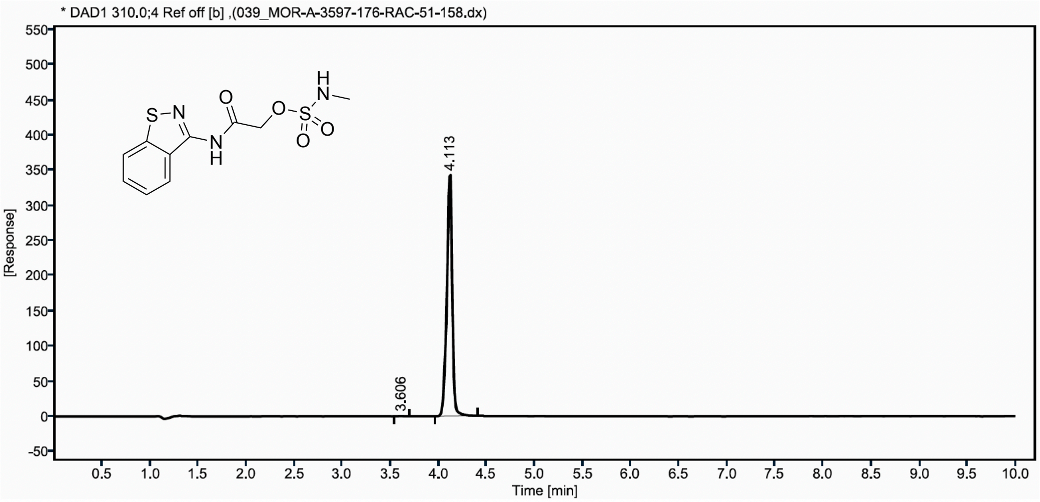

Sr.No.	RT	Area	Height	% Area
1	3.61	2	0	0.14
2	4.11	1337	342	99.86

Figure S62. HPLC trace of **8b**

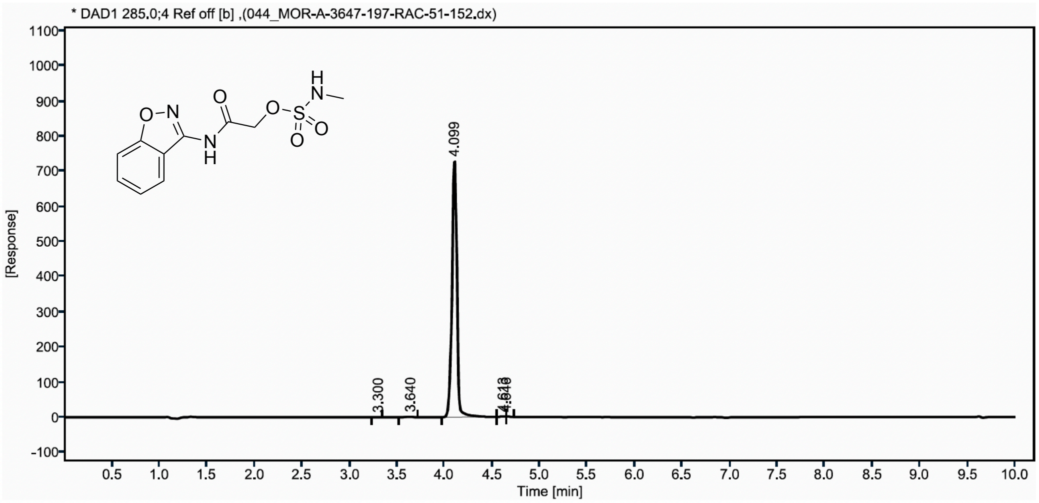

Sr.No.	RT	Area	Height	% Area
1	3.30	1	0	0.05
2	3.64	9	2	0.34
3	4.10	2663	729	99.35
4	4.61	5	1	0.17
5	4.65	3	1	0.09

Figure S63. HPLC trace of **8c**

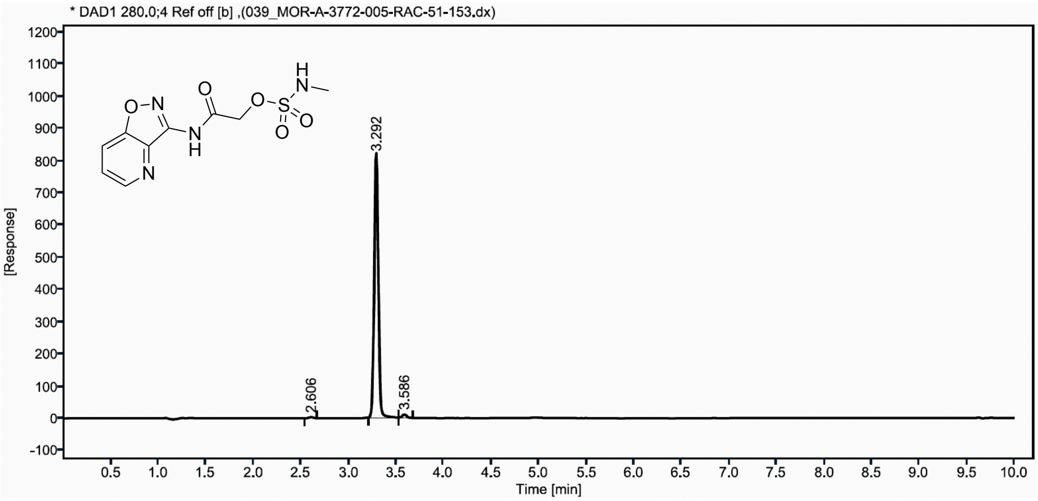

Sr.No.	RT	Area	Height	% Area
1	2.61	9	3	0.35
2	3.29	2503	813	98.36
3	3.59	33	11	1.28

Figure S64. HPLC trace of **9a**

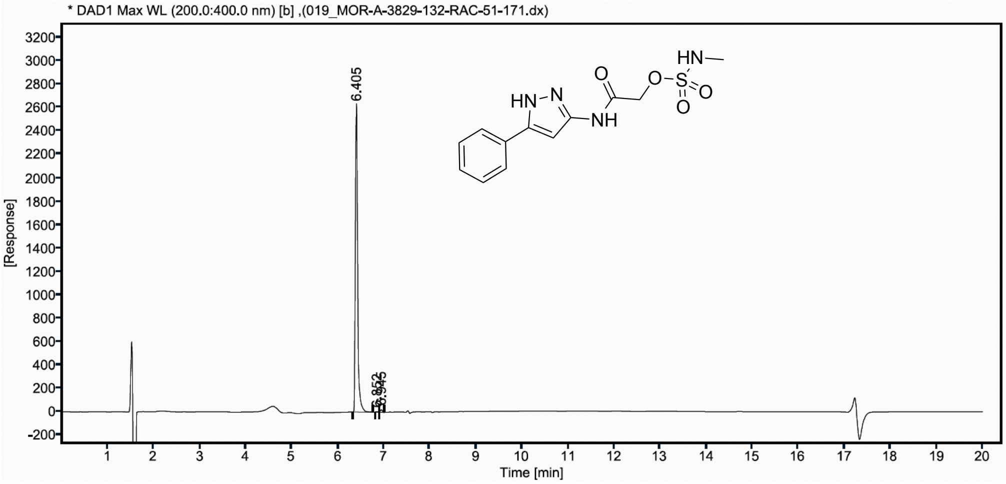

Sr.No.	RT	Area	Height	% Area
1	6.41	8877	2622	99.17
2	6.85	9	3	0.10
3	6.94	65	22	0.72

Figure S65. HPLC trace of **9b**

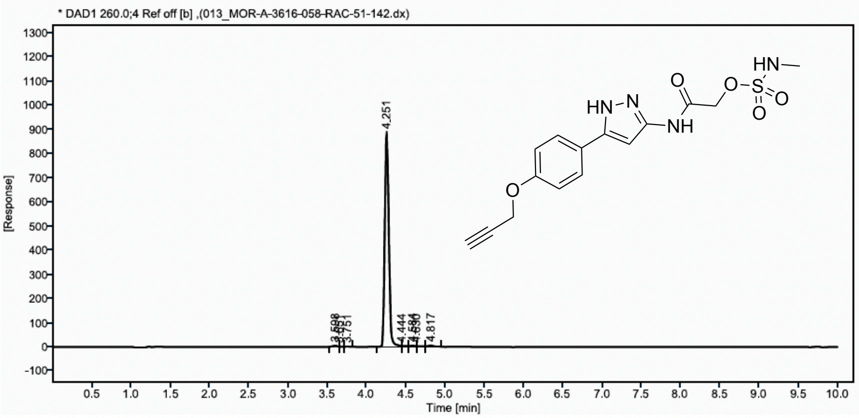

Sr.No.	RT	Area	Height	% Area
1	3.60	19	5	0.58
2	3.65	1	1	0.04
3	3.75	4	1	0.11
4	4.25	3194	886	97.20
5	4.44	13	3	0.39
6	4.58	21	5	0.63
7	4.63	11	2	0.35
8	4.82	23	5	0.71

Figure S66. HPLC trace of **9c**

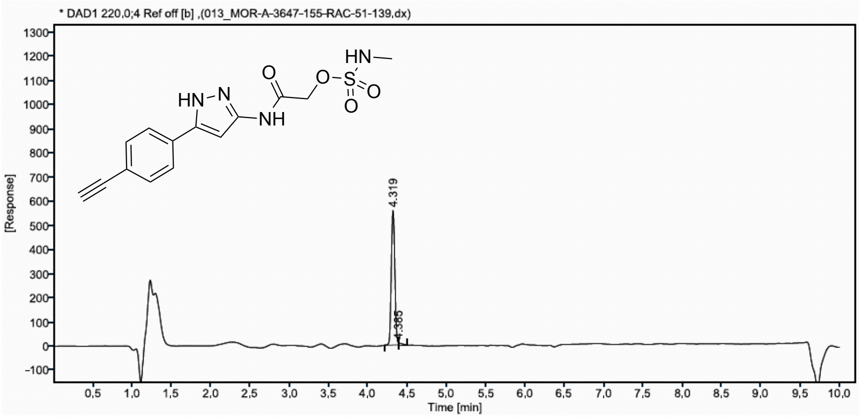

Sr.No.	RT	Area	Height	% Area
1	4.32	1723	556	98.52
2	4.39	26	8	1.48
